# Resolvins, Protectins, and Maresins: DHA-Derived Specialized Pro-Resolving Mediators, Biosynthetic Pathways, Synthetic Approaches, and Their Role in Inflammation

**DOI:** 10.3390/molecules27051677

**Published:** 2022-03-03

**Authors:** Inês Ferreira, Filipa Falcato, Narcisa Bandarra, Amélia P. Rauter

**Affiliations:** 1 Centro de Química Estrutural, Institute of Molecular Sciences, Faculdade de Ciências, Universidade de Lisboa, Ed. C8, Piso 5, Campo Grande, 1749-016 Lisboa, Portugal; fc53396@alunos.fc.ul.pt; 2 Division of Aquaculture, Upgrading and Bioprospecting, Portuguese Institute of the Sea and Atmosphere, Rua Alfredo Magalhães Ramalho, 6, 1495-006 Lisboa, Portugal; f.falcato@campus.fct.unl.pt; 3 Interdisciplinary Centre of Marine and Environmental Research (CIIMAR), University of Porto, Rua dos Bragas 289, 4050-123 Porto, Portugal

**Keywords:** marine sources, DHA, SPMs, resolvins, protectins, maresins, synthetic approaches, anti-inflammatory activity

## Abstract

Marine organisms are an important source of natural products with unique and diverse chemical structures that may hold the key for the development of novel drugs. Docosahexaenoic acid (DHA) is an omega-3 fatty acid marine natural product playing a crucial regulatory role in the resolution of inflammation and acting as a precursor for the biosynthesis of the anti-inflammatory specialized pro-resolving mediators (SPMs) resolvins, protectins, and maresins. These metabolites exert many beneficial actions including neuroprotection, anti-hypertension, or anti-tumorigenesis. As dysregulation of SPMs is associated with diseases of prolonged inflammation, the disclosure of their bioactivities may be correlated with anti-inflammatory and pro-resolving capabilities, offering new targets for drug design. The availability of these SPMs from natural resources is very low, but the evaluation of their pharmacological properties requires their access in larger amounts, as achieved by synthetic routes. In this report, the first review of the total organic syntheses carried out for resolvins, protectins, and maresins is presented. Recently, it was proposed that DHA-derived pro-resolving mediators play a key role in the treatment of COVID-19. In this work we also review the current evidence on the structures, biosynthesis, and functional and new-found roles of these novel lipid mediators of disease resolution.

## 1. Introduction

The oceans cover more than two-thirds of the earth’s surface, and it is estimated that the marine environment hosts most of the organisms living on earth. The number of marine species that inhabit the oceans is not known yet, although it is estimated that marine species include about 1–2 million of total earth species. Due to its phenomenal biodiversity, the marine world is the most valuable natural resource, and it is a very challenging, competitive, and aggressive environment that demands organisms’ adaptation to this unique habitat and the subsequent production of quite specific and potent biologically active compounds [1,2].

In the last decade, the marine environment has attracted the attention of researchers; however, oceans, as a source of novel marine drugs, remain quite unexplored. It is estimated that more than 10,000 marine natural products (MNPs), with therapeutic potential for healthcare, have been extracted and isolated from bacteria, sponges, algae, bryozoans, cnidarians, tunicates, molluscs, fungi, and other marine organisms. These compounds, mostly primary and secondary metabolites, demonstrated a unique and diverse chemical structure that may hold the key for the development of novel drug hits and leads. These compounds demonstrated significant biological activities including anticancer, anticoagulant, antiviral, antibacterial, antidiabetic, antihypertensive, antioxidant, anti-inflammatory, antimalarial, and antituberculosis properties [3]. Geographical locations can define different secondary metabolites and their concentration due to environmental factors such as salinity, temperature, pollution levels, light intensity, and predation balance [4,5]. Among the compounds that demonstrated significant biological activities are peptides isolated from fish, algal polysaccharides, carotenoids and phenolic compounds from shellfish and seaweeds, and omega-3 fatty acids from fish oil [6].

Omega-3 fatty acids are polyunsaturated fatty acids (PUFA) and have a vast nutraceutical potential with beneficial effects against a diversity of diseases (Table 1).

**Scheme 1:**
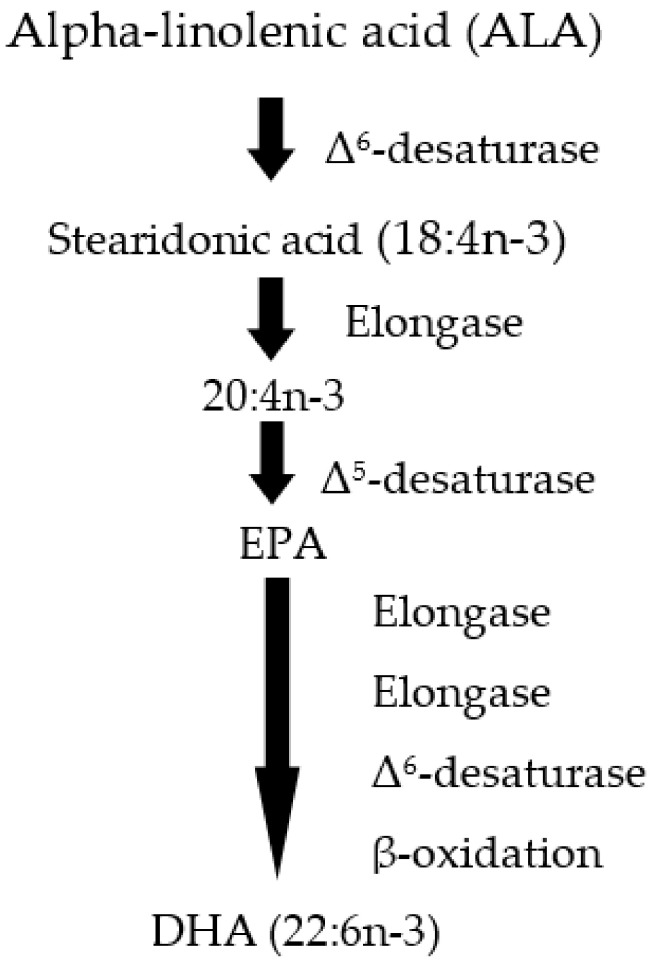
Pathway of alpha-linolenic and conversion to DHA.

Amongst omega-3 PUFA, docosahexaenoic acid (DHA, 22:6n-3) is particularly important, and despite the availability of desaturase and elongase enzymes required for the biosynthesis of DHA (Scheme 1), its synthesis is minimal in humans [14], and DHA must be obtained from dietary sources, particularly from seafood such as mackerel, salmon, herring, tuna, sardines, and products derived from seafood [15,16] or seaweeds [17].

Omega-3 fatty acids, including DHA, are mainly found esterified with glycerol as triacylglycerols (TAG), as components of phospholipid structure (PL), as cholesterol esters (CE), and as free fatty acids (FFA) [18].

**Figure 1:**
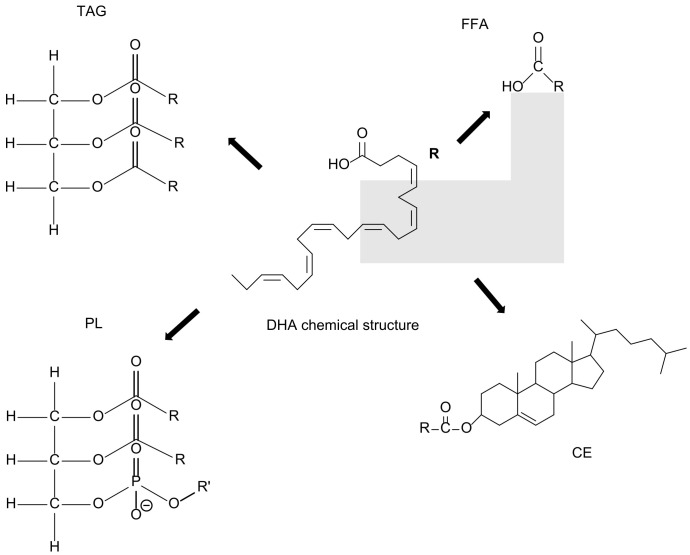
DHA chemical structure and DHA esters found in marine organisms: TAG, triacylglycerols; PL, phospholipid with R’ = serine, choline, and ethanolamine; FFA, free fatty acid; CE, cholesterol ester.

DHA has a long and highly unsaturated chain with 22 carbons and 6 double bonds (Figure 1).

DHA is essential for the growth, maintenance, and development of normal brain and visual function. It is a structural constituent of cell membranes and has a significant influence on cellular behaviour, fluidity, permeability, anti-inflammatory processes, and neuronal signalling [19]. Several studies also indicate that n-3 PUFA are essential for proper foetal development, where DHA plays a crucial role for optimal vision, cognitive development, and behaviour [20,21]. In order to prevent deficiencies associated with a low DHA intake, the WHO recommended a daily intake of DHA + EPA (eicosapentaenoic acids) at 250 mg for adult [22].

Based on a work developed by Cardoso et al. [14,18], the highest content of DHA can be found in chub mackerel (2128 mg/100 g), Atlantic salmon (1773 mg/100 g), or European eel (3447 mg/100 g). Additionally, microalgae *Thraustochytrium aureum* ATCC 34,304 are rich in DHA (6590 mg/100 g of dry matter) [14].

## 2. From DHA to SPMs and Their Functional Role

**Figure 2:**
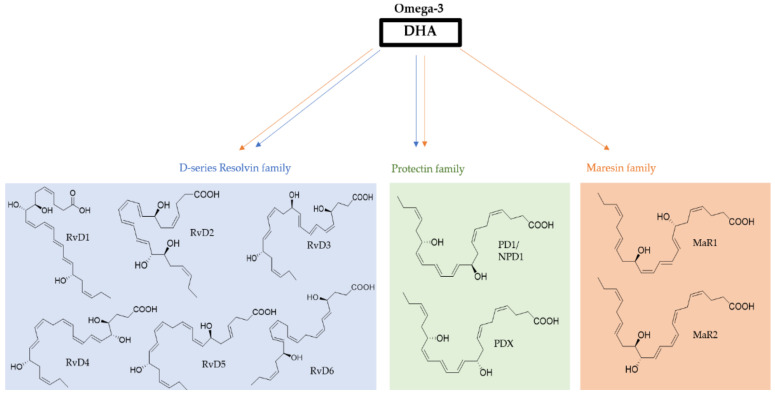
Structure of the SPMs D-series resolvins, protectins, and maresins, which are metabolites derived from DHA.

DHA is a substrate for cyclooxygenases (COXs) and lipoxygenases (LOXs), being enzymatically oxidized to mono-, di-, and tri-hydroxyDHA (HDHAs), oxidized to epoxides, oxidized to oxo-DHA metabolites, or oxidized to neuroprostanes, among other compound families. HDHAs include the specialized pro-resolving mediators (SPMs) or docosanoids, comprising D-series resolvins (RvD), protectins (PD), and maresins (MaRs) (Figure 2), which are available at sites of acute inflammation, acting in a specific tissue [23], and potent lipid mediators, with benefits of the prevention and treatment of several diseases. HDHAs have shown benefits of neuroprotection, benefits against the risks of cardiovascular diseases, and anti-tumour properties, among others [24].

Four types of SPMs have been classified depending on the precursor molecule that they derive from and the enzyme implicated in metabolic cascade: lipoxins (LXs), RvD and RvE, PD, and MaRs. Lipoxins (LXs) result from a biosynthetic pathway of omega-6 fatty acid: araquidonic acid. LXs were the first SPM to be described and studied due to their essential role in various inflammatory diseases [25]. Resolvins from EPA (E-resolvins) and from DHA (D-resolvins) and protectins and maresins from DHA are structurally distinct from LXs resulting from a different metabolic pathway from omega-3 fatty acids.

SPMs are active in subnanomolar doses in between the picogram and nanogram levels, promoting stereospecific activation of cellular receptors on diverse cells of immune system and organ-specific protectors. SPMs have special functions and direct action on inflammation control and resolution phase. They can act on immune cells such as neutrophils and macrophages, endothelial and epithelial cells, and lymphocytes [26,27].

Given the influence of uncontrolled inflammation to diverse human pathologies, the identification of endogenous control mechanisms in acute inflammatory response has become essential. Inflammation reaction is an essential protective mechanism for preserving health and normally is well-organized and controlled, leading to a quick response and normal tissue structure repair. However, if the wound healing response is dysregulated, it can be characterized by a weak inflammatory response that can lead to tissue destruction by harmful stimuli or chronic unresolved inflammation. This event may culminate in diverse pathologies such as arthritis, cardiovascular disease, neurodegenerative diseases such as Alzheimer’s and Parkinson′s, asthma, cancer, and autoimmune diseases [27,28]. Following microbial infection or tissue injury, the release of pro-inflammatory lipid mediators is among the first signalling events, promoting the recruitment of neutrophils (polymorphonuclear leukocytes (PMN)) [29]. Although the acute inflammatory response is protective, when an excess of PMN congregates or swarms in tissues, it can lead to tissue damage that ultimately amplifies inflammation, causing a wide range of acute, chronic, and systemic inflammatory disorders [27,29,30,31]. Thus, it becomes crucial to understand the underlying mechanisms and specialized mediators involved in the resolution process.

Known as immunoresolvents and recognized by their potent stereoselective, pro-resolving, and anti-inflammatory actions, SPMs can stimulate the resolution of inflammation without immune suppression [27,32,33,34]. Each family of SPM will promote the recruitment and activation of monocytes and will block neutrophil recruitment, which will lead to both the resolution of inflammation and homeostasis through the combined actions of these mediators [29]. SPMs are enzymatically produced in human body fluids and organs, being involved mainly in the resolution of inflammation, wound healing, and neuroprotection. The use of SPMs can reduce the treatment with antibiotics against infection and is a new approach to the development of alternative and effective therapies for several diseases [24,35]. The identification of resolvins, protectins, and maresins is of great importance, as these potent lipid mediators provide the first molecular basis for many of the health benefits attributed to DHA. However, for many of them, the specific molecular mechanisms remain unclear [29,36,37,38]. Thus, disclosure of the biosynthetic pathways for conversion of DHA to SPMs, along with their structural elucidation and bioactivities evaluation, is crucial to the understanding of inflammation resolution. By promoting the resolution of inflammation, clearance of microbes, reduction in pain, and promotion of tissue regeneration via specific cellular and molecular mechanisms, they have gained the attention of the scientific community [39]. The anti-inflammatory and pro-resolution functions of SPMs are attained by binding to specialized G-protein-coupled receptors (GPCRs). Between these GPCRs are GPR32, FPR2/ALX, GPR18 for resolvins, RORα and ALX/FPR2 for maresins, and GPR84 and GPR120 for protectins [35,40,41].

**Scheme 2:**
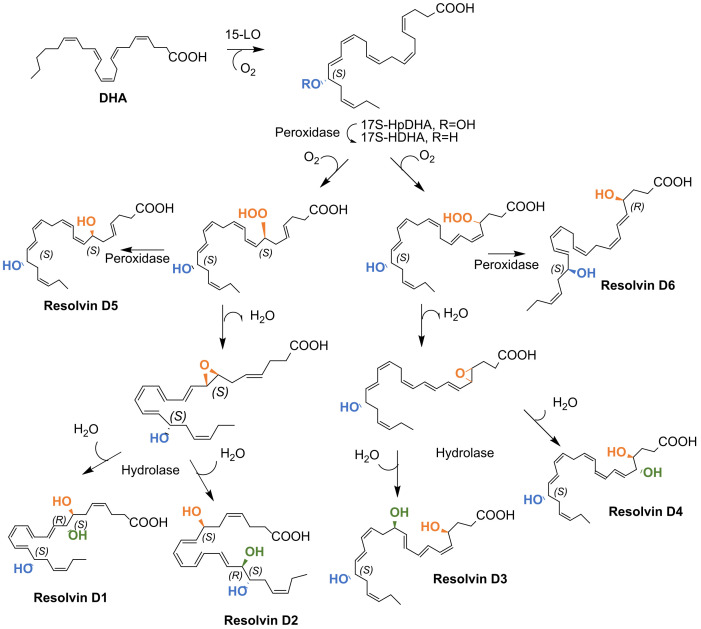
D-series resolvins biosynthesis by LOX mechanism.

**Scheme 3:**
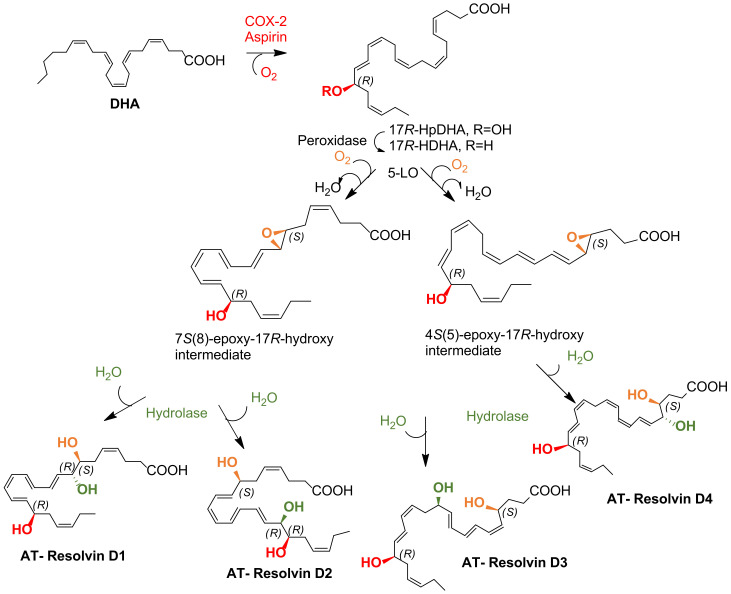
D-series resolvins biosynthesis by aspirin-triggered COX-2.

Some of the molecular and cellular effects of DHA-derived SPMs are summarized in Table 2. Within the resolvins family, RvD1, RvD2, RvD3, RvD4, RvD5, and RvD6 were identified as well as the series of lipid mediators’ aspirin-triggered forms (AT-RvD), differing in their stereochemistry (Scheme 2 and Scheme 3), as well as protectins PD1 and MaR1 and MaR2.

In the next sections, the biosynthesis and the total organic synthesis of these pro-resolving mediators are presented and discussed.

## 3. Resolvins from DHA

Pro-resolving oxylipins biosynthesized from DHA, commonly known as Rvs, are primarily anti-inflammatory in nature [65]. Resolvins have strong ability to respond for regulation of pro-inflammation and actively stimulate resolution via monocyte/macrophage uptake of debris, apoptotic PMN, and by clearing microbes [36,66,67]. Chunrong and co-workers [35] revealed that these metabolites can have even more potent anti-inflammatory and pro-resolving activities than their precursors. The Rvs derived from DHA are Rvs of D-series and were reported as neuroprotective agents, exerting their protective effect against cell injury-induced oxidative stress [38,68,69]. The six identified resolvins and the aspirin-triggered forms are biosynthesized by aspirin-triggered cyclooxygenase-2 [24,26,35] (see Section 3.1). Due to the stereospecific activation of cellular receptors, each SPM carries defined biological functions with cell type- and organ-specific properties [70]. Recently, the beneficial roles of resolvins against diabetes and kidney disease were revised [71]. In various experimental kidney disease models, RvD1 was found to restore kidney tubule function, inhibit NF- κB and IL-6 activation [72], increase regulatory T cells, and attenuate tubular injury [73] and to attenuate fibrosis, fibroblast proliferation, and collagen deposition in an experimental model of unilateral ureteral obstruction [74]. Resolvins also suppress tumour growth and enhance cancer therapy [75].

### 3.1. Biosynthesis of D-Series and Aspirin-Dependent D-Series Resolvins

D-Series Rvs biosynthesis occurs in mononuclear cells, such as macrophages and neutrophils, endothelial cells, fibroblasts, and monocytes (under inflammatory conditions), and in vascular cells and tissues [35,76]. There are two pathways to biosynthesize RvDs: by the lipoxygenase (LOX) mechanism (Scheme 2) or through the aspirin-triggered cyclooxygenase-2 (COX-2) pathway (Scheme 3) [27,29,35].

The biosynthetic conversion of DHA to D-series Rv (RvD1-RvD6) is illustrated in Scheme 3. The initial step proceeds via lipoxygenation of DHA at carbon 17 catalysed by 15-lipoxygenase (15-LO) to give (17S,4Z,7Z,10Z,13Z,15E,19Z)-17-hydroperoxydocosahexaenoic acid (abbreviated as 17S-HpDHA), which is transformed into the corresponding 17S-hydroxyDHA (17S-HDHA) by a peroxidase. A second lipoxygenation by 5-LO at carbon 7 is followed by catalysis with a peroxidase to give RvD5. Dehydration affords an intermediate epoxide, which when hydrolysis-catalysed by a hydrolase gives RvD1 and RvD2. Lipoxygenation at carbon 4 catalysed by 5-LO gives a hydroperoxide, which generates RvD6 under peroxidase catalysis or is dehydrated to form a 4,5-epoxide, which in turn, under hydrolase catalysis, gives RvD3 and RvD4. The steps covering 5-lipoxygenation and epoxide formation can occur within a single cell type or via transcellular biosynthesis [29].

Alternatively, RvDs biosynthesis can be initiated by the aspirin-dependent acetylated COX-2 enzyme to give the 17R-series RvDs. In fact, acetylated COX-2 in the presence of aspirin acts as a modified dioxygenase by introducing an oxygen molecule with opposite stereochemistry [29]. The RvDs biosynthesized through aspirin and acetylated COX-2 also undergo lipid oxidation catalysed by 15-LO and 5-LO, epoxidation, and hydrolysis processes, forming AT-RvDs (Scheme 3).

The biosynthetic pathway of AT-RvDs could partly explain some anti-inflammatory effects of aspirin since COX-2 in its presence biosynthesizes metabolically more stable SPM epimers, with pro-resolving and anti-inflammatory activities [29,77]. As shown in Scheme 2 and Scheme 3, RvDs and AT-RvDs differ in their *R*/*S* configuration of each OH group containing chirality centre. AT-RvDs are equally potent anti-inflammatory agents, but the “*R*” isomers have longer biological half-lives and have a higher resistance to the metabolic inactivation by oxidoreductases [59,78].

### 3.2. Total Synthesis of D-Series Resolvins

The availability of resolvins from natural resources is low, but they can be generated in vitro via one-pot single enzyme incubation [43,79], although in vivo RvD1 requires two lipooxygenation steps. However, for the evaluation of their biological and pharmacological properties, it is of great importance to have an easy access to these compounds, which has challenged scientists to develop synthetic routes towards resolvins.

#### 3.2.1. Resolvin D1

**Scheme 4:**
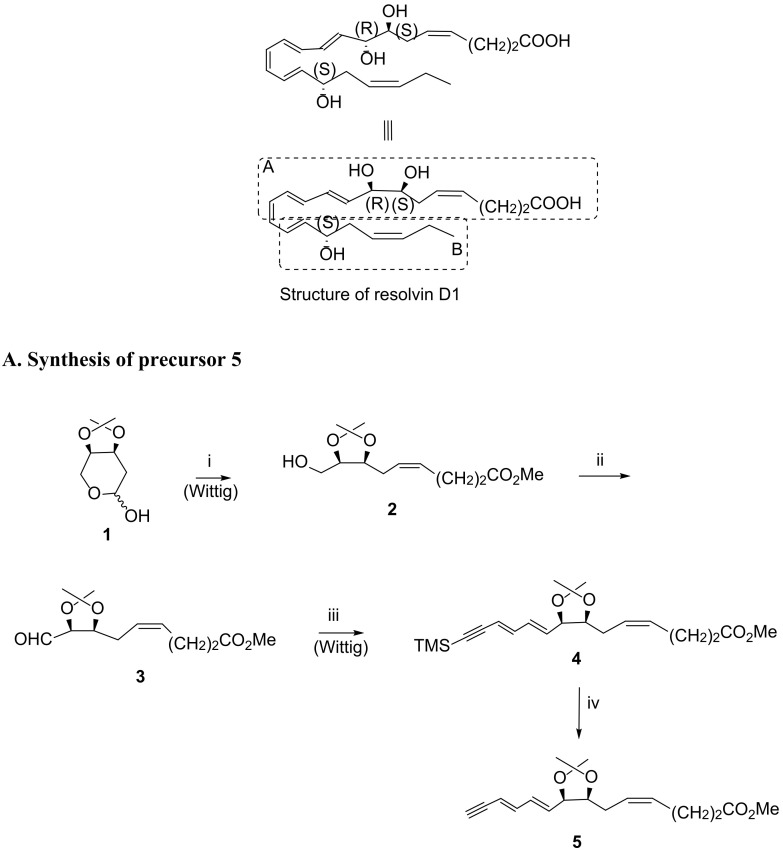

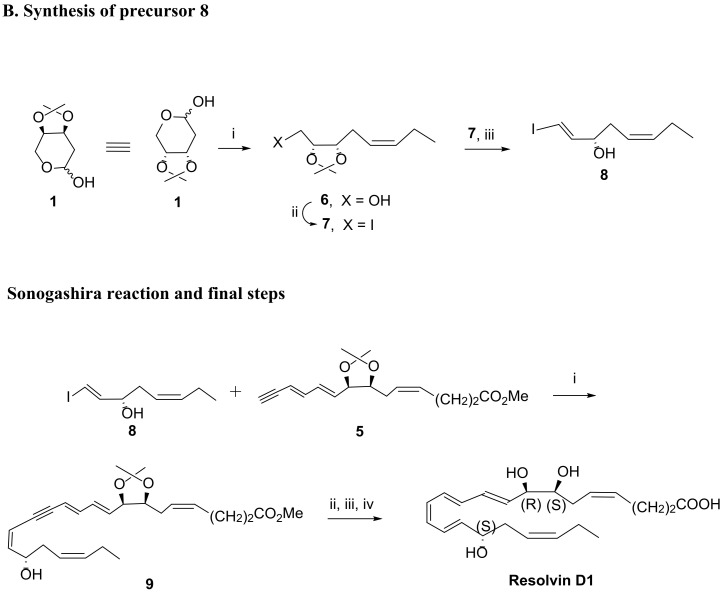
A RvD1 total synthesis as reported in 2012 by Rodríguez and Spur [80] (**A**). Synthesis of compound **5**. Reagents and conditions: i. KN(TMS)_2_, MeO_2_C-(CH_2_)_2_CH_2_PPh_3_^+^ Br^−^, THF, −70 °C to rt, 43%; ii. Dess–Martin periodinane, CH_2_Cl_2,_ rt, 84%; iii. 1.TMS-C≡C-CH = CHCH_2_PPh_3_^+^ Br^−^, *n*BuLi, THF, −70 °C to 0 °C; 2. I_2_, benzene, rt, 87%; iv. KF^·^2H_2_O, 18-crown-6, DMF, rt, 88%. (**B**) RvD1 total synthesis as described in 2012 by Rodríguez and Spur [80]. (**B**). Synthesis of compound **8**. Reagents and conditions: i. CH_3_CH_2_CH_2_PPh_3_^+^ Br^−^, NaN(TMS)_2_, Et_2_O, −78 °C to rt, 60%; ii. I_2_, Ph_3_P, imidazole, toluene, 60 °C, 82%; iii. LDA, THF, −78 °C, 40%; Synthesis of RvD1 via Sonogashira reaction. Reagents and conditions: i. Pd(PPh_3_)_4_, CuI, piperidine, benzene, rt, 86%; ii. CH_3_COCl cat., CH_3_OH, 0 °C, 90%; iii. Zn(Cu/Ag), CH_3_OH, H_2_O, 50 °C, 5 h, 67%; iv. LiOH 1N, CH_3_OH, H_2_O, 0 °C, then sat. NaH_2_PO_4_, 94%.

The first total synthesis of RvD1 was carried out by Serhan et al., via a Sonogashira reaction, whose studies also covered RvD1 natural product structure assignment [29,79]. In 2012, Rodríguez and Spur [80], inspired by Serhan et al. reports [29,79], reported an elegant and successful approach, also based on the Sonogashira reaction, by reacting the alkyne component containing part A and the iodide containing part B, as depicted in Scheme 4 [80]. The starting material was 2-deoxy-3,4-O-isopropylidene-d-erythro-pentopyranose (**1**), used twice to install a double bond through Wittig olefination (Scheme 4A,B). The synthesis of the cis-isomer **2** was achieved in 43% yield by reaction of **1** with the phosphorane resulting from the treatment with base of [(ethoxycarbonyl)propyl] triphenylphosphonium bromide. Dess–Martin periodinane oxidation of the primary alcohol gave the aldehyde, which was again submitted to another Wittig reaction with the appropriate phosphorane, followed by cis-trans isomerism with iodine/benzene to afford the trans-trans diene isomer **4** in 80% yield. Cleavage of the trimethylsilyl group with potassium fluoride afforded the triple bond required for the Sonogashira reaction, in which precursor **5** reacts with the iodoester **8**, installing RvD1 parent structure, as depicted in Scheme 4. The synthesis of **8** was carried out by Wittig reaction of **1** with propylenetriphenylphosphorane, followed by treatment with butyl lithium and acid work-up, providing **8** in 19.7% overall yield from **1** (Scheme 4A). The Sonogashira reaction gave compound **9** in high yield, which was submitted to isopropylidene cleavage in acid medium generated by CH_3_COCl in methanol. After reduction of the triple bond with Zn (Cu/Ag) and hydrolysis of the ester with lithium hydroxide, the RvD1 was obtained in 56.7% yield from precursor **9** (yield over the last three steps).

Recently, in 2019, Kobayashi et al. [81] reported the synthesis of RvD1 also using a Sonogashira reaction but changing the reaction partners. In this approach, the iodoester **10** reacted with the alcohol with the terminal triple bond to afford RvD1 after reduction with Zinc, deprotection with *tert*-butyl ammonium fluoride, and ester hydrolysis, in overall yield of 72.5%.

The preparation of the iodoester **10** was accomplished in fifteen steps in an overall yield of 2.3% starting from butyrolactone. After the first seven steps to obtain the epoxide **12** [81], Swern oxidation of the primary alcohol was followed by treatment with base to generate epoxide ring opening and elimination, giving the unsaturated aldehyde **13**, the hydroxy group of which was then protected with the *tert*-butyldimethylsilyl (TBS) group. Then, reaction with TMSC(Li)N_2_ [82,83] afforded the *trans*-enyne **15** in very good yield. Hydrozirconation with Cp_2_Zr(H)Cl, obtained in situ from Cp_2_ZrCl_2_ and diisobutylaluminium hydride (DIBAL) [84], followed by iodinolysis of the zirconium species formed, gave **16** in reasonable yield (75%). Removal of silyl groups with *tert*-butylammonium fluoride (TBAF) and resilylation to have all free hydroxy groups protected with the TBS group was followed by regioselective deprotection with piridinium *p*-toluenesulfonate (PPTS) to afford the free primary hydroxy group. Swern oxidation gave the aldehyde **19**, which was submitted to the Wittig reaction, providing the target iodoester **10**, one of the Sonogashira reaction partners.

**Scheme 5:**
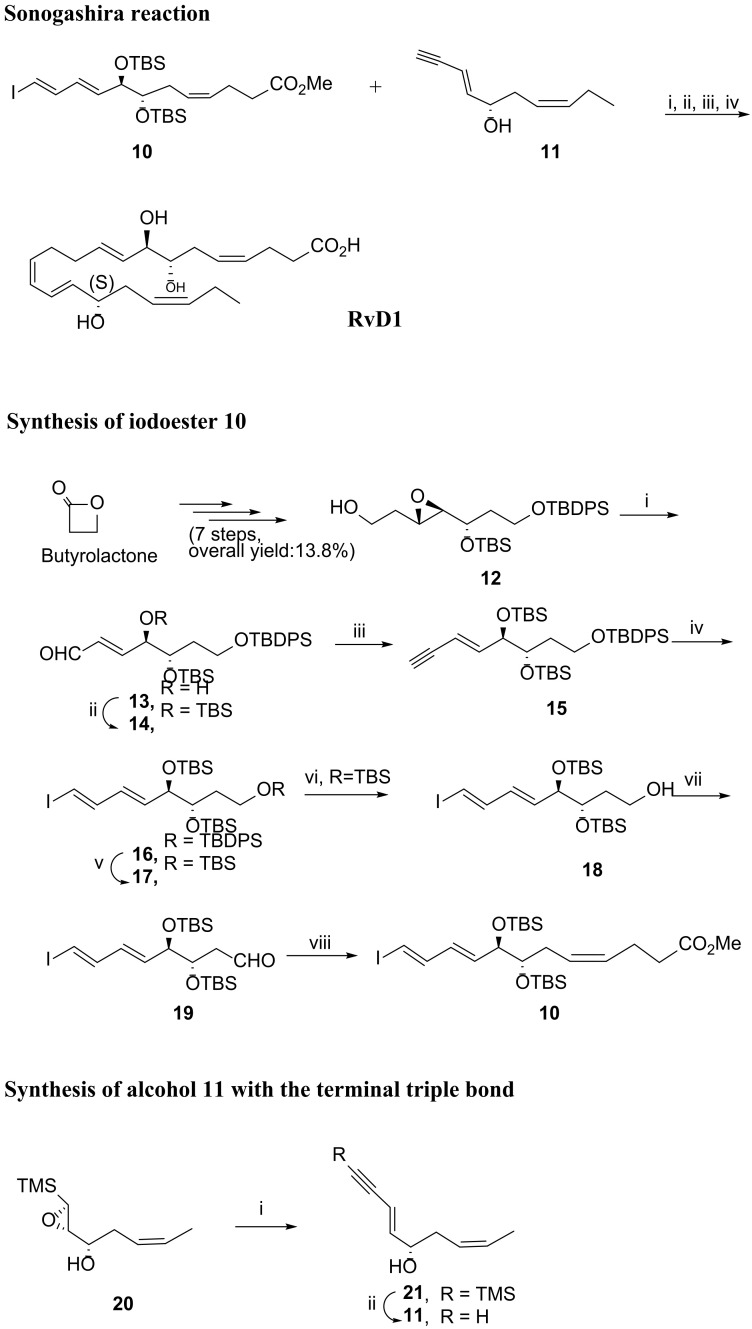
Synthesis of RvD1, as reported by Kobayashi et al. [81]. Reagents and conditions. Sonogashira reaction: i. Pd (PPh_3_)_4_, CuI, *t*BuNH_2_, rt, 15 min; ii. TBAF, THF, rt, 2 h, 78% over two steps; iii. Zn (Cu/Ag) (excess), MeOH/H_2_O (1:1), rt, 5 h, 93%; iv. LiOH, aq. MeOH, 42%; Synthesis of iodoester **10**: i. 1. (COCl)_2_, DMSO, CH_2_Cl_2_; 2. Net_3_, −78 °C to 0 °C, 83%; ii. TBSOTf, 2,6-lutidine, 92%; iii. TMSCHN_2_, LDA, THF, −78 °C to rt, 89%; iv. 1. Cp_2_ZrCl_2_, DIBAL (1:1), THF, −78°C to rt; 2. I_2_, −78 °C to rt, 75%; v. 1. TBAF, THF, 2 h, rt; 2. TBSOTf, 2,6-lutidine, 79%; vi. PPTS (1.1. equiv.), MeOH/CH_2_Cl_2_, rt, 3 h, 50%; vii. (COCl)_2_, DMSO; then Net_3_; viii. Br—Ph_3_P^+^CH_2_CH_2_CH_2_CO_2_Me, NaHMDS, THF, −78 °C to 0 °C, 73% over the two steps; Synthesis of alcohol **11**: i. TMSC≡CH, *n*BuLi, THF/HMPA (1:1), rt, 4 h; ii. K_2_CO_3_, MeOH, rt, 1 h, 59% over the two steps.

The synthesis of the Sonogashira reaction partner **11** was carried out starting from epoxide **20** [81], which reacted first with lithium acetylide and then with potassium carbonate for trimethylsilyl cleavage, giving **11** in 59% overall yield (Scheme 5).

#### 3.2.2. Resolvin D2

**Scheme 6:**
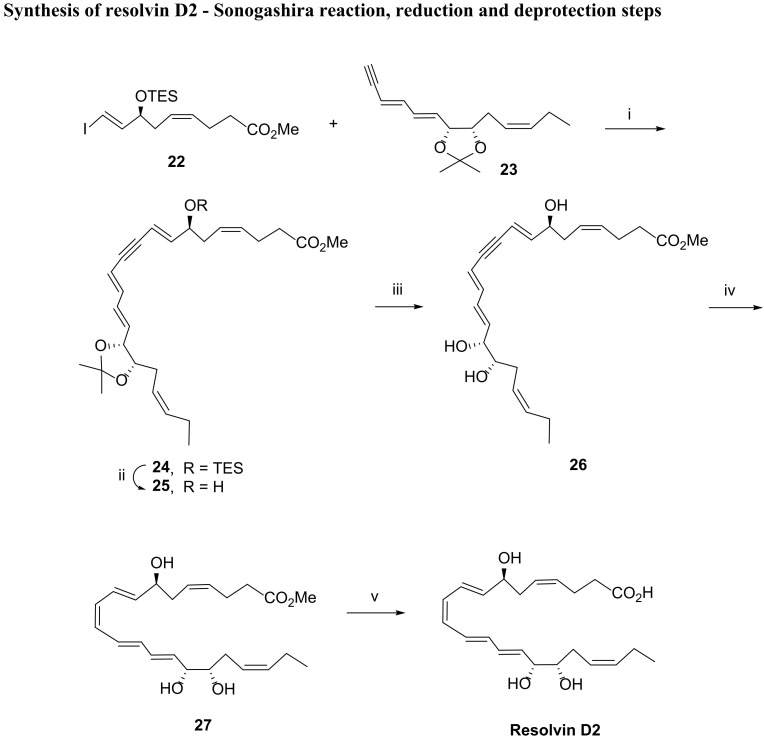
Synthesis of resolvin D2 via Sonogashira reaction. Reagents and conditions: i. Pd (PPh_3_)_4_, CuI,n-PrNH_2_,benzene, rt; ii. pyridinium, *p*-toluenesulfonate, CH_3_OH, rt; iii. 1N HCl, CH_3_OH/H_2_O, rt; iv. Zn (Cu/Ag), aq CH_3_OH, 40 °C; v. 1N LiOH, THF, 0 °C, then EtOAc, satd NaH_2_PO_4_.

The first total synthesis of RvD2 was reported by Rodríguez and Spur in 2004 [85]. The key step is again a Sonogashira reaction, in which the iodoester **22** reacts with the terminal triple bond of the isopropylidene protected *cis*-diol **23** (Scheme 6). After deprotection of the triethylsilyl (TES) group with PPTS and acid hydrolysis of the isopropylidene, the triol **26** is reduced with Zn (Cu/Ag), a stereospecific reaction affording the ester **27** in 70% yield, the hydrolysis of which provided resolvin D2.

**Scheme 7:**
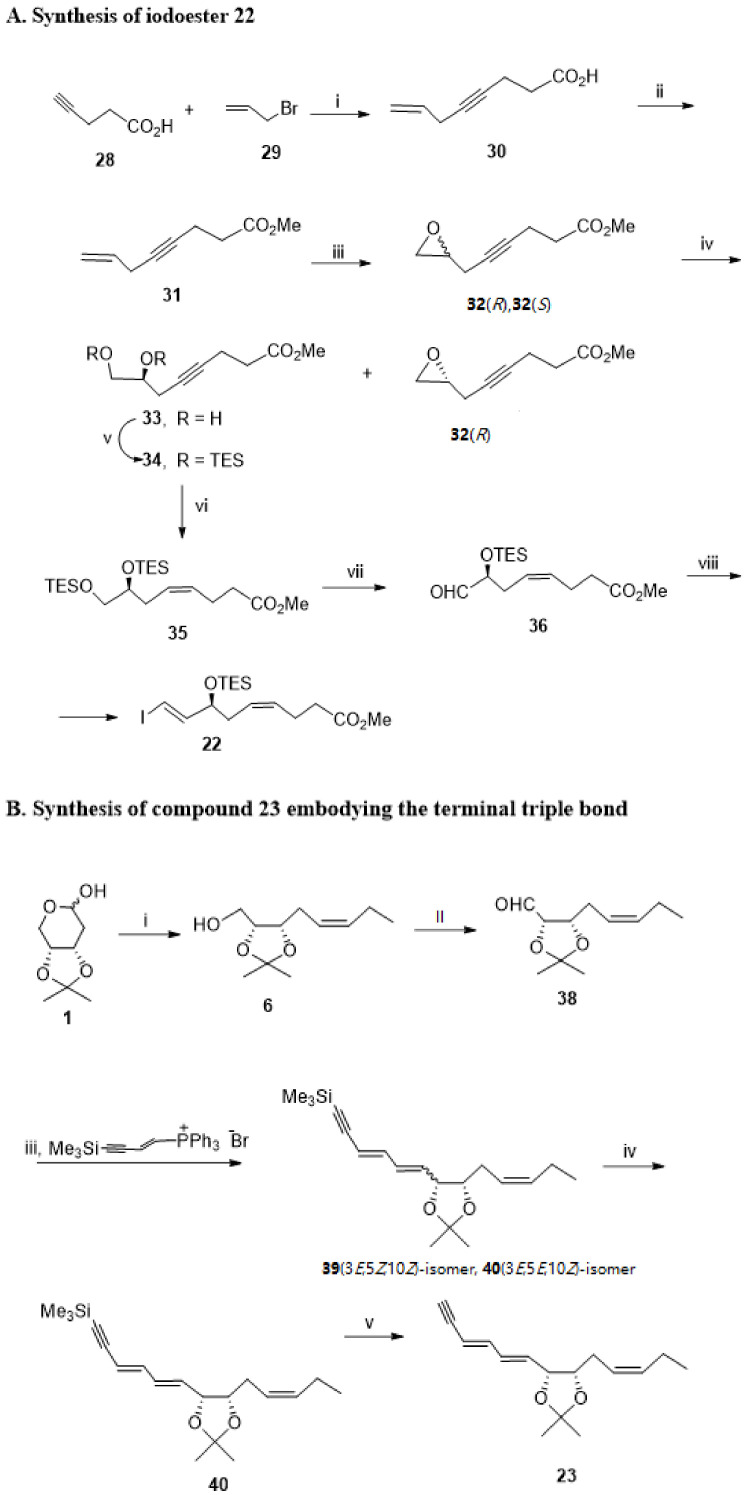
(**A**) Synthesis of iodoester **22**. Reagents and conditions: i. MeMgBr, CuBr-Me_2_S, THF, 0 °C to rt; ii. 10% TMSCl, MeOH, 2,2-dimethoxypropane, rt; iii. *m*CPBA, NaHCO_3_, CH_2_Cl_2_, 0 °C to rt; iv. (*R*,*R*)-(salen)CO(III)(OAc) catalyst, Et_2_O/H_2_O, 0 °C to rt; v. TESCl, imidazole, Et_3_N, DMF, 0 °C to rt, 84%; vi. Lindlar catalyst, H_2_, Et_3_N, hexane, quantitative yield; vii. (COCl)_2_, DMSO, CH_2_Cl_2_, Et_3_N, −78 °C to rt, 54%; viii. CrCl_2_, CHI_3_, THF, 0 °C, 50%. (**B**) Synthesis of compound **23** embodying the terminal triple bond. Reagents and conditions: i. CH_3_CH_2_CH_2_Ph_3_P^+^ Br^−^, NaN(TMS)_2_, Et_2_O, −78 °C to 0 °C; ii. PCC, NaOAc, CH_2_Cl_2,_ 70%; iii. BuLi, THF, −78 °C to 0 °C; iv. I_2_, benzene, rt; v. KF, 18-crown-6, DMF, rt.

Starting with the pent-4-ynoic acid **28**, that forms a dimagnesium complex with methylmagnesium bromide, alkylation proceeds via reaction with allyl bromide **29** in the presence of catalytic CuBr-Me_2_S, furnishing oct-7-en-4-ynoic acid **30**, esterified in situ to give **31** (Scheme 7A). Epoxidation with *m*-chloroperbenzoic acid (*m*CPBA) provided epoxide racemic mixture **32**. The hydrolytic kinetic resolution with water in the presence of 5% (*R*,*R*)-salen-Co (III)(OAc) catalyst in diethyl ether furnished diol **33** with >94% enantiomeric excess, separated by column chromatography from epoxide **32(*R*)**. Both hydroxy groups were then protected by reaction with triethylsilyl chloride, followed by reduction of the triple bond using the Lindlar catalyst to afford quantitatively the ester **35** with a *cis*-double bond. Chemoselective Swern oxidation furnished aldehyde **36** in 54% yield, which was subjected to Takai olefination to give **22** in 50% yield.

The preparation of Sonogashira reagent **23** was carried out starting with Wittig of the sugar **1** with the phosphorane resulting from propyltriphenylphosphonium bromide treatment with the base, furnishing the unsaturated primary alcohol **6** with a *cis*-double bond (Scheme 7B). Oxidation with pyridinium chlorochromate (PCC) afforded aldehyde **38** isolated in 70% yield. Wittig reaction with the phosphorane, formed by butyl lithium treatment of [(2*E*)-5-trimethylsilylpent-2-en-4-ynyl] triphenylphosphonium bromide, gave a mixture of isomers differing in the (*Z*), and the (*E*)-configuration of the double bond formed, but with a catalytic amount of iodine in benzene, isomerization occurred, and only the desired Wittig product **40** was obtained. Cleavage of TMS was accomplished with potassium fluoride in dimethylformamide and 5% of 18-crown-6 ether to give the target compound **23** in high yield.

**Scheme 8:**
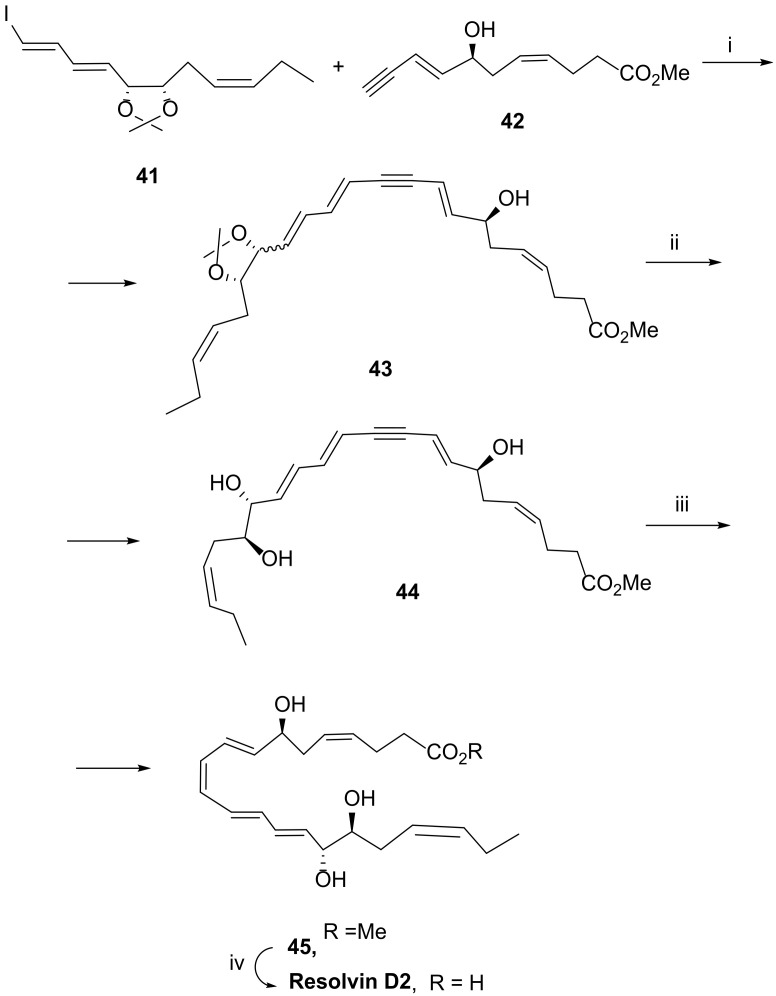
Synthesis of resolvin D2, as reported by Rizzacasa et al. [86]. Reagents and conditions: i. Ph_3_PdCl_2_, CuI, Et_3_N, 0 °C to rt, 83%; ii. MeOH, HCl, rt, 85%; iii. Zn (Cu/Ag), MeOH, H_2_O, 76%; iv. LiOH, 1M, THF, 0 °C, 81%.

In 2013, Rizzacasa et al. [86] disclosed the total synthesis of RvD2 by a convenient and high yield approach, also based on a Sonogashira coupling. By coupling the hydroxyester **41** embodying the triple bond with the iodide **42**, catalysed by (Ph3P)2PdCl2/CuI, intermediate **43** was formed in high yield (Scheme 8). Isopropylidene hydrolysis gave triol **44** also in high yield, and reduction of the triple bond with Zn (Cu/Ag) afforded the ester **45** in 76% yield. Ester hydrolysis with lithium hydroxide 1M furnished resolvin D2 with the overall yield of 43.4%. This methodology has the advantage of reducing the number of steps from five to four, when compared with the first total synthesis previously described.

**Scheme 9:**
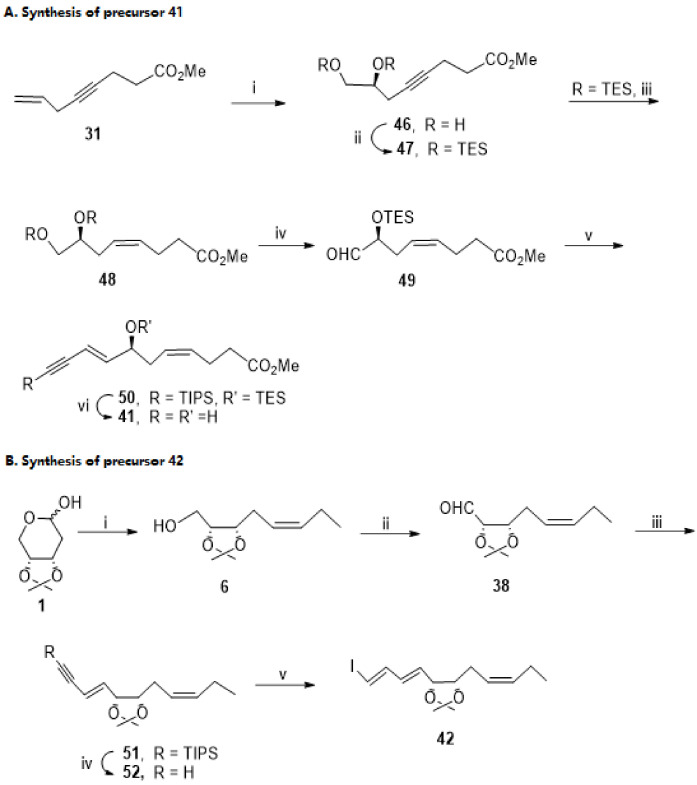
Preparation of precursors **41** (**A**) and **42** (**B**), as reported by Rizzacasa et al. [86]. Reagents and conditions: (**A**). i. Admix-α, MeSO_2_NH_2_, tBuOH, H_2_O, 69%; ii. TESCl, imidazole, 93%; iii. Ni (OAc)_2_.4H_2_O, NaBH_4_, EDA, MeOH, 73%; iv. (COCl)_2_, DMSO, Et_3_N, CH_2_Cl_2_,78%; v. 1. TIPS-≡-CH_2_PPh_3_^+^Br^−^, LiHMDS, −78 °C, then addition of **38**, −40 °C, (3*E*,8*Z*)-isomer 49% and (3*Z*,8*Z*)-isomer 12%; vi. TBAF. (**B**). CH_3_CH_2_CH_2_PPh_3_^+^ Br^−^, NaHMDS, −78 °C to 0 °C, 74%; ii. DMP, 93%; iii. TIPS-≡-CH_2_PPh_3_^+^ Br^−^, nBuLi, THF, −40 °C to 0 °C, (1*E*,3*E*,8*Z*)-isomer 57%, (1*Z*,3*E*,8*Z*)-isomer 26%; iv. TBAF, 73%; v. 1. ZrCp_2_Cl_2_, iBu_2_AlH, THF, 0 °C; 2. I_2_, −78 °C, 64%.

The preparation of substrates **41** and **42** is described in Scheme 9A,B, respectively. The synthesis of compound **41** started by asymmetric dihydroxylation of compound **31** [85] catalysed by ADmix-α, furnishing diol **46** in 69% yield. After protection of the hydroxy groups by reaction with triethylsilyl chloride, partial reduction of the triple bond succeeded with sodium borohydride and nickel acetate in ethylenediamine (EDA), an efficient system for producing pure (*Z*)-alkenes, which afforded the unsaturated ester **48** in 73% yield. In agreement with the findings of Rodríguez et al. [87], Swern oxidation of **48** afforded the aldehyde **49** in high yield, which reacted then with the Wittig reagent to give as major product the methyl ester **50** with the (4*Z*,8*E*)-4,8-diene functionality. Desilylation with TBAF gave **41** in 15.2% overall yield starting from compound **31**.

Synthesis of **42** (Scheme 9B) started from compound **6**, previously reported by Rodríguez and Spur (Scheme 7B) [85]. Oxidation with Dess–Martin periodinane (DMP) afforded the aldehyde **38** in very high yield (93%), when compared with the yield of 70%, previously reported when oxidation was carried out with PCC [85]. Introduction of the enyne moiety by Wittig reaction gave the required 3(*E*)-isomer **51** as the major product, in 57% isolated yield. Desilylation with TBAF was followed by stereoselective reduction of the triple bond with the system ZrCp2Cl_2_ and DIBAL, furnishing product **42**, containing the required trans-iododiene moiety, in good yield.

#### 3.2.3. Resolvin D3

**Scheme 10:**
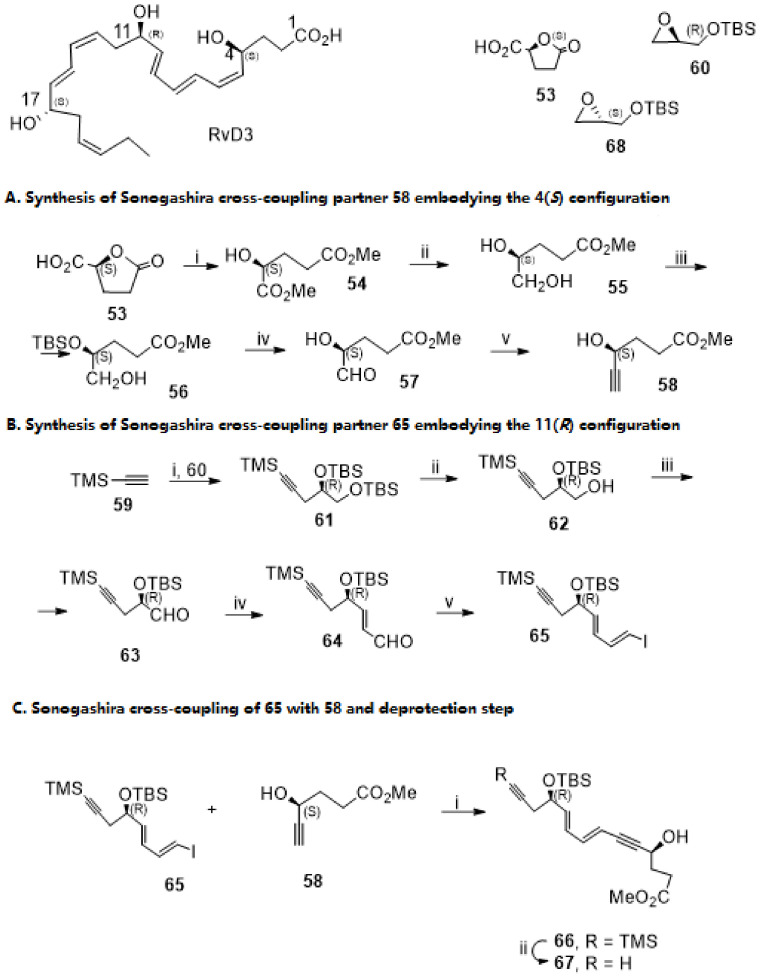
Synthesis of Sonogashira cross-coupling partners **58** and **65**, Sonogashira reaction, and deprotection step to afford ester **67**. Reagents and conditions: (**A**). i. MeOH, HCl, cat. 96%; ii. BH_3_.DMS, NaBH_4_, THF, 10 °C, 86%; iii. 1. TBSCl, imidazole, DMAP, CH_2_Cl_2_; 2. CSA, CH_2_Cl_2_/MeOH, 57%; iv. (COCl)_2_, DMSO, Et_3_N, CH_2_Cl_2_, −78 °C, 98%; v. 1. PPh_3_, CBr_4_, CH_2_Cl_2_, 0 °C; 2. LDA, THF, −78 °C, 62%. (**B**). i.nBuLi, BF_3_.Et_2_O, THF, −78 °C; 2. 60; 3. TBSCl, imidazole, DMAP, CH_2_Cl_2_, 63%; ii. CSA, rt, CH_2_Cl_2_/MeOH, 77%; iii. (COCl)_2_, DMSO, Et_3_N, CH_2_Cl_2_, −78 °C, 90%; iv. Ph_3_P = CHCHO, PhMe, 2 h, 83%; v. CHI_3_, CrCl_2_, THF, 0 °C, 84%; (**C**). i. Pd(PPh_3_)_4_, CuI cat., Et_3_N, PhH, rt, 92%; ii. Na_2_CO_3_, MeOH, rt, 91%.

**Scheme 11:**
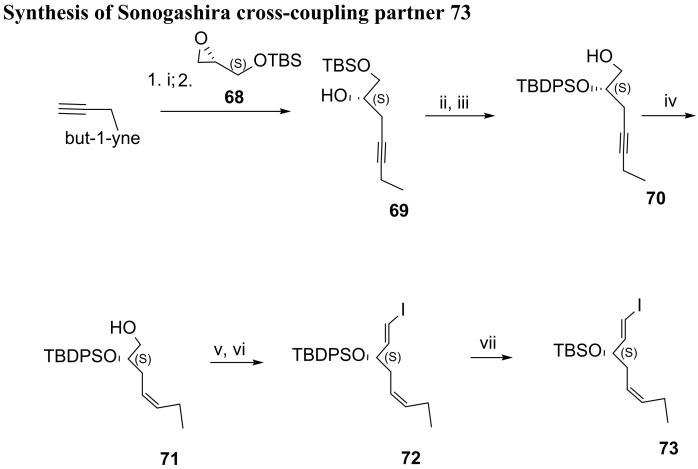
Synthesis of Sonogashira cross-coupling partner **73**. Reagents and conditions: i. nBuLi, BF_3_ Et_2_O, THF, −78 °C; ii. **68**, 73%; ii. TBDPSCl, imidazole, DMAP, CH_2_Cl_2_, 89%; iii. CSA, CH_2_Cl_2_/MeOH, rt, 97%; iv. H_2_/Lindlar cat., EtOAc, rt, 94%; v. (COCl)_2_, DMSO, Et_3_N, CH_2_Cl_2_, −78 °C, 90%; vi. CHI_3_, CrCl_2_, THF, 0 °C, 59%; vii. 1. TBAF, THF, rt; 2. TBSOTf, lutidine, CH_2_Cl_2_, rt, 69%.

**Scheme 12:**
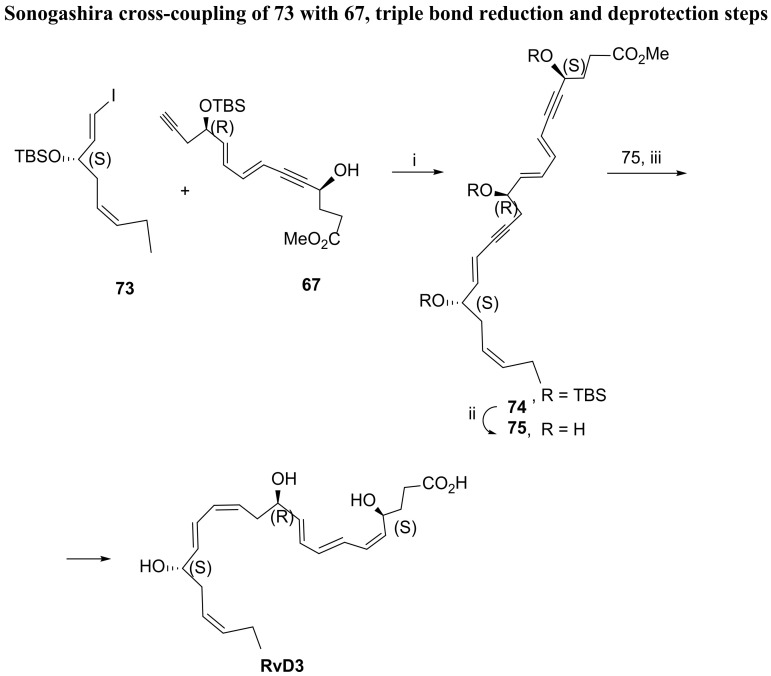
Synthesis of RvD3—the second Sonogashira reaction. i. Pd (PPh_3_)_4_, CuI cat., Et_3_N, PhH, rt, 92%; ii. TBAF, THF, 27%; iii. 1. Zn/Cu/Ag, MeOH/H_2_O; 2. NaOH, H_2_O, MeOH, 22% (over the two steps).

In 2013, Petasis and co-workers developed the first total synthesis of RvD3. The convergent and stereocontrolled synthetic pathway (Scheme 10, Scheme 11 and Scheme 12) involves two Sonogashira cross-coupling reactions introducing key triple bonds that are stereoselectively reduced with the mild reagent Zn (Cu/Ag) to generate triene and diene moieties in the final steps, aiming at preventing *Z*/*E* isomerization. Starting from carboxy-γ-butyrolactone **53** (Scheme 10A) [which (*S*)-configuration generates the 4(*S*) configuration of RvD3], lactone ring opening, and esterification, followed by regioselective reduction with borane dimethyl sulfide in the presence of catalytic sodium borohydride, gave diol **55** in high yield. Silylation of the free hydroxy groups was followed by regioselective desilylation of the primary position with camphorsulfonic acid (CSA). After Swern oxidation of the primary alcohol in very high yield, Corey–Fuchs homologation using LDA as base gave compound **58** in 62% yield, which is the cross-coupling partner of the first Sonogashira reaction. Its coupling partner is compound **65**, prepared from epoxide **60** with (*R*)-configuration, which is maintained along the synthetic pathway, generating the 11(*R*)-chirality centre of RvD3. Nucleophilic attack of **60** with TMS-acetylide anion followed by silylation of the formed hydroxy group gave compound **61**. Regioselective desilylation with CSA was followed by Swern oxidation to give the aldehyde **63**, which reacted with formylmethylenetriphenylphosphorane, affording the *trans*-isomer **64** in 86% yield. Finally, Takai olefination of **64** provided **65** as the major product from a 9:1 mixture of the *E/Z* isomers, embodying the iododiene moiety required for the Sonogashira reaction and the terminal alkyne for the second Sonogashira reaction, which is required for the total synthesis of RvD3.

Sonogashira cross-coupling of **65** and **58** was catalysed by Pd (Ph_3_)_4_/cat CuI in triethylamine and benzene, affording a very high yield (92%) of enantiomerically pure compound **66**, which was desilylated with sodium carbonate in methanol, giving **67** also in very high yield.

The preparation of the **73** cross-coupling partner for the second Sonogashira reaction used, as starting material, the epoxide enantiomer **68** (Scheme 11), which reacted with lithiated but-1-yne attacking the less hindered carbon of the epoxide and proving alcohol **69** with the required (*S*) configuration. Protection of the secondary alcohol with tert-butyldiphenylsilyl (TBDPS) group was followed by TBS cleavage to afford the primary alcohol **70**. Hydrogenation with Lindlar catalyst resulted in a stereoselective reduction of the triple bond, giving alcohol **71** in very high yield, embodying a cis-double bond. Swern oxidation gave the aldehyde precursor in 90%, and the stereoselective olefination with CH_3_I/CrCl_2_, the so-called Takai olefination, afforded precursor **72** in good yield. The protecting group TBDPS was removed, and the secondary position protected with TBS to give **73**, because in the final assembly of RvD3, removal of TBDPS with TBAF led to product decomposition. The Sonogashira cross-coupling of **73** and **67** succeeded in very high yield (Scheme 12). TBS removal also succeeded in giving the expected deprotected product **75**, although in low yield (27%), and the target molecule was prepared by triple bond reduction with Zn (Cu/Ag) and ester hydrolysis, in 22% yield over the last two steps.

**Scheme 13:**
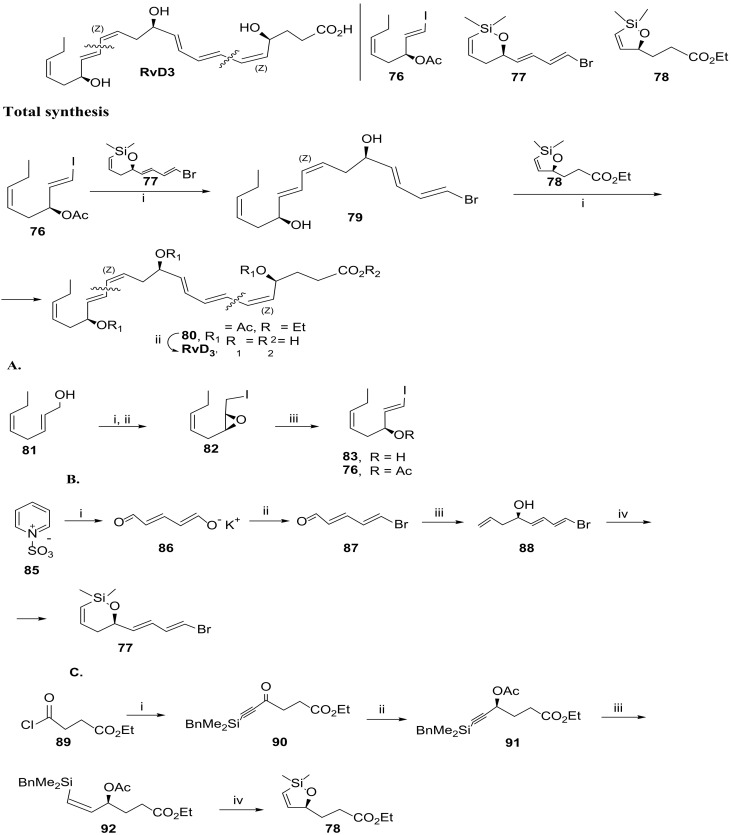
Synthesis of RvD3, as reported by Anderson and co-workers [88], and preparation of the building blocks **76** (**A**), **77** (**B**), and **78** (**C**). Reagents and conditions: Total synthesis: i. 1. Pd(dba)_2_, TBAF, THF, 16 h; 2. Ac_2_O, py, CH_2_Cl_2_, 56% for **79** and 32% for **80**, both over the two steps ii. LiOH, THF/H_2_O (1:1), 99%; (**A**). Preparation of **76**: i. 1. Ti(O-*i*Pr)_4_, (l)-(+)-DIPT, TBHP, 3 Å MS, CH_2_Cl_2_, −20 °C, 75%, 92% ee; 2. I_2_, PPh_3_, imidazole, CH_2_Cl_2_, 53%; iii. Ac_2_O, DMAP, Et_3_N, CH_2_Cl_2_, 0 °C to rt, 2 h, 93%; (**B**). Preparation of **77**. i.KOH, 58% [X8]; ii. Ph_3_PB_r2,_ 50% (isolated yield) [89]; iii. 1.CH_2_=CHCH_2_MgCl, THF, 0 °C; 2. Ti(O-*i*Pr)_4_, (l)-(+)-DIPT, TBHP, 3 Å MS, CH_2_Cl_2_, −20 °C, 97%ee, 38% yield over the two steps; iv. 1. CH_2_ = CHSiMe_2_Cl, Et_3_N, CH_2_Cl_2_; 2. Schrock catalyst, PhH, 87%; Preparation of **78**: i. BnMe_2_SiC≡CMgBr, 57%; ii. 1. RuCl(*S, S*)TsDPEN, *i*PrOH; 2. Ac_2_O cat., DMAP, Et_3_N, CH_2_Cl_2_, 88%, 97% ee; iii. Pd/CaCO_3_, quinoline, H_2_, PhMe-cyclohexene (10:1), 83%; iv. TBAF, THF, quantitative yield.

In 2020, Anderson and co-workers [88] developed an alternative synthetic pathway, leading to a very original and concise synthesis of RvD3, by applying a new approach based on the stereoselectivity of the cross-coupling of cyclic alkenylsiloxanes, used to specifically control the stereochemistry of the installed (*Z*)-double bonds (Scheme 13). They conceived a tail-to-head strategy with the building blocks **76**, the RvD3 tail fragment for the cross-coupling with the six-membered ring siloxane **77**, the middle fragment that reacts with **78**, and the five-membered ring siloxane B head fragment to obtain protected RvD3, which, in one single step, by reaction with aqueous lithium hydroxide, provided RvD3 in very high yield. Synthesis of the building block **76** (Scheme 13) started with the Sharpless epoxidation of the allyl alcohol **81** to afford the correct configuration of the epoxide, glowed by Garegg iodination of the primary alcohol. Base mediated the regioselective epoxide ring opening, and elimination afforded alcohol **83**, embodying the desired (*E*)-iodoalkene moiety. Synthesis of **77** started with (2*E*,4*E*)-5-bromopenta-2,4-dienal, prepared by treatment of pyridinium-1-sulfonate sodium hydroxide to afford glutaconaldehyde potassium salt, followed by bromination with triphenylphosphonium bromide [89] (Scheme 13). Addition of allyl magnesium bromide, and Sharpless resolution gave the enantio-enriched (97% ee) alcohol **88** in 38% yield. Reaction with dimethylvinylsilyl chloride gave the intermediate ether for the successful and high yield ring-closing metathesis mediated by the Schrock catalyst. The preparation of **78** started with the reaction of the commercially available acid chloride **89** with [(benzyldimethylsilyl)ethynyl] magnesium bromide to afford ketone **90** in 57% yield. Stereoselective reduction by Noyori asymmetric transfer hydrogenation gave the alcohol in 97% yield and 99% enantiomer excess, which was then esterified with acetic anhydride and dimethylaminopyridine (DMAP). Stereoselective reduction of the triple bond to the (*Z*)-double bond was challenging and succeeded in 83% yield (*Z/E*:20/1). Debenzylation, deacetylation, and cyclization occurred by treatment with TBAF, which afforded the building block **78** in quantitative yield.

#### 3.2.4. Resolvin D4

**Scheme 14:**
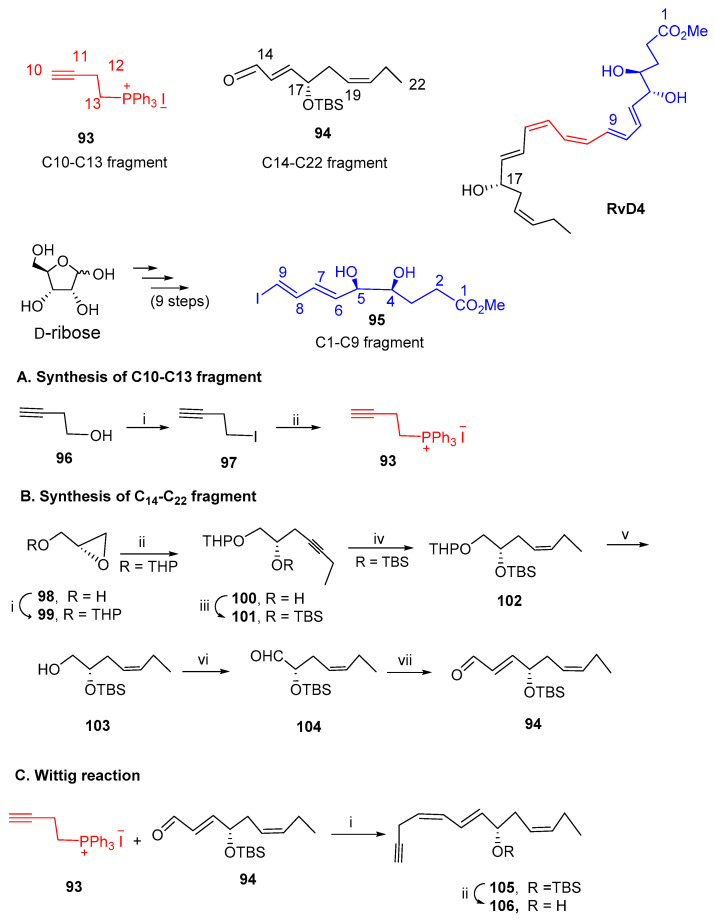
Preparation of the building blocks for the synthesis of RvD4, as reported by Serhan et al. [90]. (**A**). Synthesis of C10–C13 fragment; (**B**). Preparation of C14-C22 fragment; (**C**). Synthesis of Sonogashira cross-coupling partner **106**. Reagents and conditions: (**A**). i. I_2_, imidazole, PPh_3_, CH_2_Cl_2_,4h; ii. PPh_3_, MeCN, reflux, 12 h; (**B**). i. cat.PTSA-H_2_O, DHP, 5 min; ii. But-1-yne, nBuLi, BF_3_.Et_2_O, THF, 3 h; iv. TBSCl, imidazole, DMF, 3 h; Ni (OAc)_2_.4H2O, NaBH_4_, H_2_N(CH_2_)_2_NH_2_, H_2_, EtOH, 12 h; v. MgBr_2_, Et_2_O, 4 h; vi. (COCl)_2_, DMSO, Et_3_N, CH_2_Cl_2_, 0.5 h; vii. Ph_3_P=CHCHO, Net_3_, PhH, 12 h; (**C**). i. NaHMDS, THF, −78 °C, 3 h; ii. TBAF, THF, 40 °C, 48 h.

**Scheme 15:**
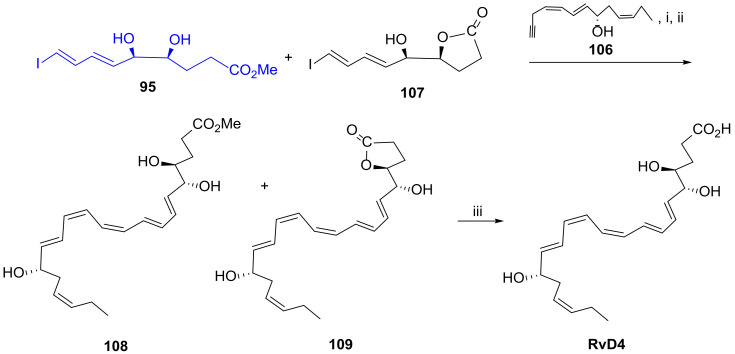
Sonogashira reaction and final steps conducting to the total synthesis of RvD4, as reported by Serhan et al. [90]. Reagents and conditions: i. Pd(PPh_3_)_4_, CuI, *n*BuNH_2_, PhH, 12 h; ii. Zn(Cu/Ag), MeOH (aq.), 40 °C, 12 h; iii. K_2_CO_3_, MeOH, 12 h.

The complete stereochemistry of RvD4 was established only in 2016 [60], and later in 2018, an organic total synthesis was reported [90], allowing multi-milligram to scale up, thus enabling commercial production of RvD4. The approach is based on assembling three building blocks, the first one consisting of C14–C22, fragment **94**, with a terminal carbonyl group which reacts with the C10–C13 fragment **93** through a Wittig olefination providing, after desilylation, the cross-coupling partner **106** embodying the terminal triple bond required for the Sonogashira reaction (Scheme 14). However, the authors found out that iodoester C1–C9 **95** was contaminated with the iodolactone **107**, a secondary product resulting from intramolecular cyclisation, which could not be separated from **95** (Scheme 15). This problem was overcome by running Sonogashira reaction with the ester and lactone mixture. After reduction with Zn (Cu/Ag) affording compounds **108** and **109**, treatment of the mixture with potassium carbonate in methanol hydrolysed both the ester and the lactone, providing RvD4 as a single product.

#### 3.2.5. Resolvin D5

**Scheme 16:**
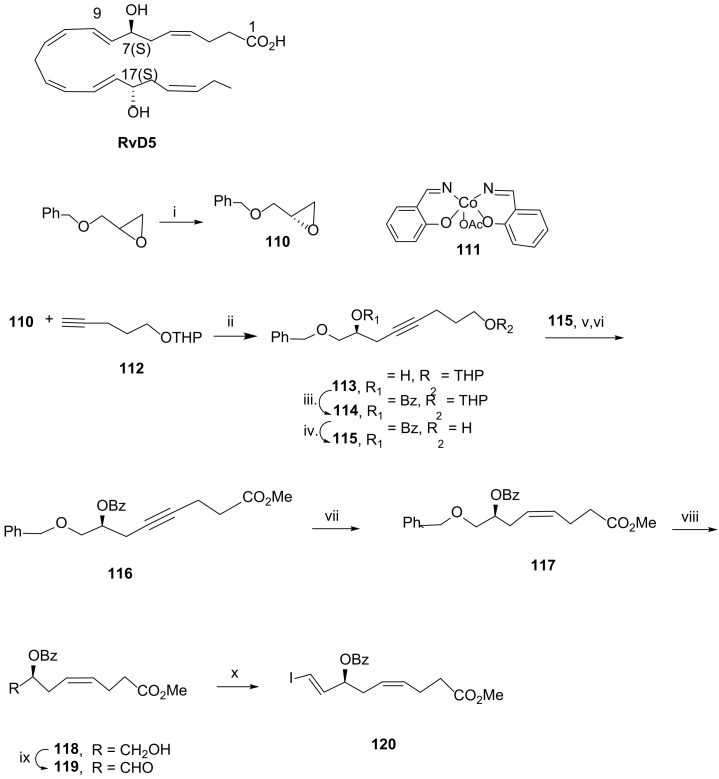
Synthesis of precursor **120** for the construction of the C1-C9 moiety of RvD5 [91]. Reagents and conditions: i. **111**, H_2_O, 0 °C to rt, 45% yield, >99% ee; ii. nBuLi, BF_3_.Et_2_O, THF, −70 °C, 85%; iii. BzCl (2 equiv), Py, 0 °C to rt, 92%; iv. PPTS cat., MeOH, rt, 88%; v. Jones reagent, acetone, 0 °C; vi. 10% TMSCl, MeOH, 2,2-dimethoxypropane, rt, 93% over the last two steps; vii. H_2_, Lindlar cat., hexane, 98%; viii. EtSH, AlCl_3_, CH_2_Cl_2_, −35 °C, 86%; ix. Py.SO_3_, DMSO, CH_2_Cl_2_, Et_3_N, 86%; x. CrCl_2_, CHI_3_, THF, rt, 52%.

**Scheme 17:**
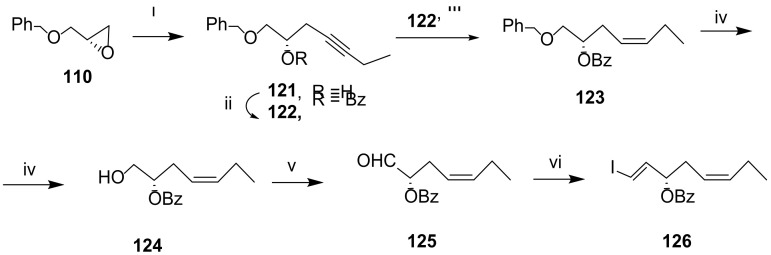
Preparation of precursor **126** for the construction of the C15-C22 moiety of RvD5 [91]. Reagents and conditions: i. HC≡CCH_2_CH_3_, *n*BuLi, BF_3_.Et_2_O, THF, −70 °C, 80%; ii. BzCl (2 equiv), Py, 0 °C to rt, 94%; iii. H_2_, Lindlar cat., hexane, 96%; iv. EtSH, AlCl_3_, CH_2_Cl_2_, −35 °C, 85%; v. Py^.^SO_3_, DMSO, CH_2_Cl_2_, Et_3_N, 84%; vi. CrCl_2_, CHI_3_, THF, rt, *trans*-vinyl iodide/*cis*-vinyl iodide (4:1).

**Scheme 18:**
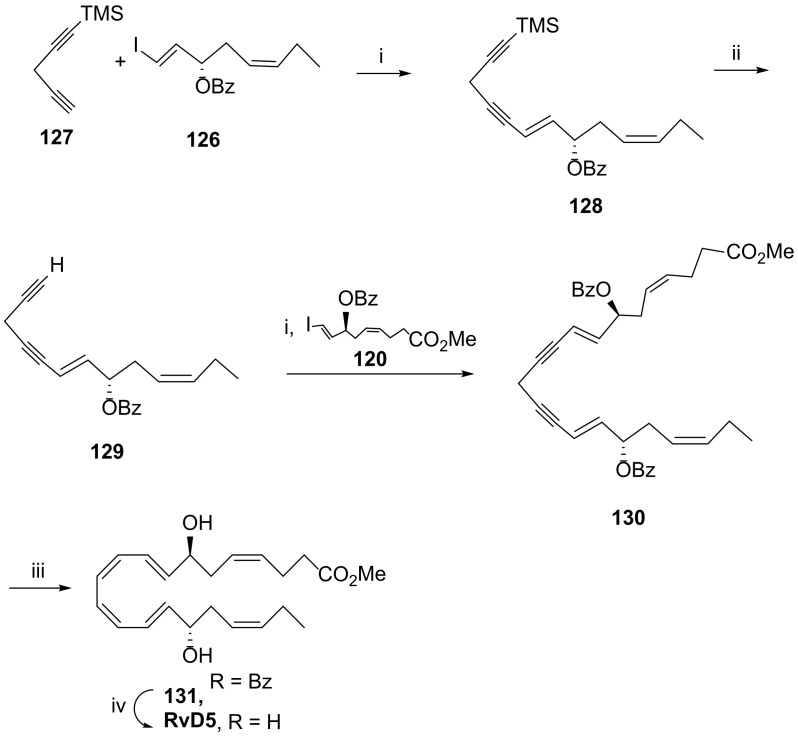
First total synthesis of RvD5 [91]. Reagents and conditions: i. Pd (PPh_3_)_4_, CuI, PrNH_2_, benzene, rt, 50% (**128**), 54% (**130**); ii. AgNO_3_, MeOH, H_2_O, NaCN; iii.H_2_, Lindlar cat., Et_3_N, hexane, 84%; iv. Lipase from *Candida rugosa*, H_2_O (CaCl_2_, NaCl), THF, 75%.

The first total synthesis of RvD5 was reported by Rodríguez and Spur in 2005 [91]. The chirality centres 7(*S*) and 17(*S*) were installed via epoxide **110**, obtained by hydrolytic kinetic resolution of the epoxide racemic mixture in the presence of water and (*R*, *R*)-salen-Co (III)(Oac) (**111**) catalyst (Scheme 16). The preparation of the precursor of RvD5 C1-C9 moiety started by nucleophilic epoxide ring opening with the alkynide resulting from treatment of **112** with butyllithium to afford the alcohol **113**, which was then protected with the benzoyl group. After cleavage of the tetrahydropyranyl group, the resulting primary alcohol **115** was oxidized with Jones reagent, and in situ esterification gave ester **116**. After Lindlar reduction, the isomer embodying the *cis* double bond was formed in very high yield (98%). Debenzylation with EtSH and aluminum trichloride gave the primary alcohol, the oxidation of which, followed by Takai olefination [92], afforded iodide **120**, one of the reaction partners to afford the total synthesis of RvD5. The synthesis of the C15-C22 precursor **126** followed a similar strategy, starting with the reaction of epoxide **110** with the anion of but-1-yne generated with butyllithium (Scheme 17). Benzoylation of the alcohol, Lindlar reduction, debenzylation, Swern oxidation, and Takai olefination afforded a mixture of the *trans*/*cis*-vinyl iodides difficult to separate, with the *trans*-isomer being the major product and present in proportion 4:1. The mixture was submitted to Sonogashira reaction with iodide **126**, but only the more reactive trans-vinyl alkene reacted to afford **128** in 50% yield. After cleavage of TMS, a second Sonogashira reaction gave the methyl ester **130**. Controlled hydrogenation with Lindlar catalyst, deactivated by Net_3_, afforded the dibenzoyl precursor **131** in good yield (Scheme 18). Ester hydrolysis and benzoyl deprotection with the lipase *Candida rugosa* succeeded in 75% yield, while attempts to use hydrolysis in mild alkaline conditions only hydrolysed the ester, and stronger basic conditions required for benzoyl deprotection led to product degradation.

**Scheme 19:**
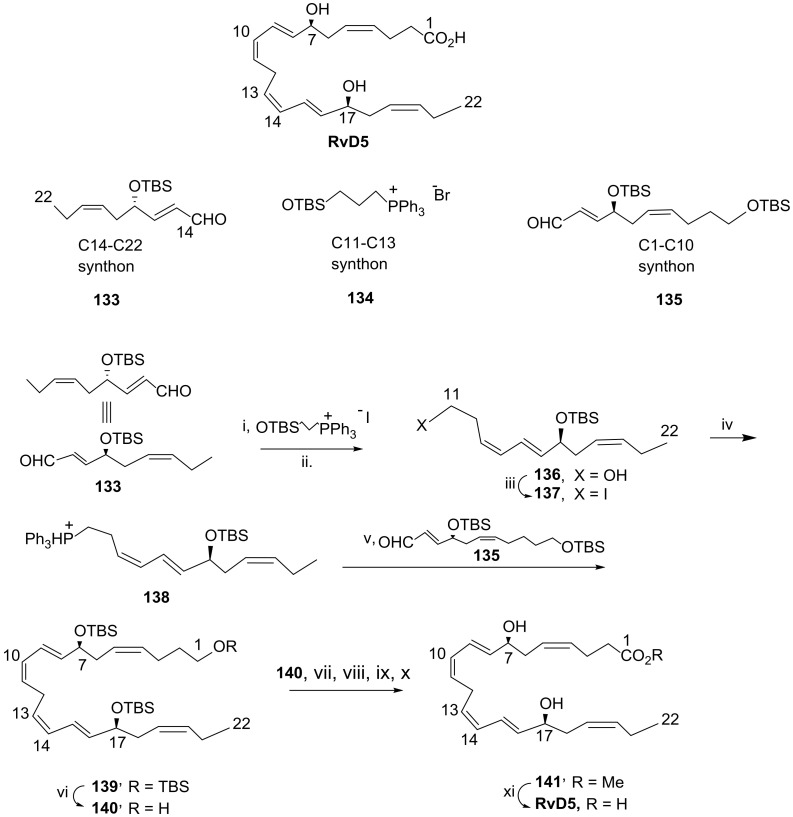
Synthesis of RvD5, as reported by Kobayashi and co-workers [93]. Reagents and conditions: i. NaHMDS, THF, −70 °C, 8 h; ii. PPTS, MeOH, rt, 77% over the two steps; iii. I_2_, Ph_3_P, imidazole, 85%; iv. Ph_3_P, MeCN, quant.; v. NaHMDS, −90 °C to 0 °C; vi. PPTS, MeOH, 53% over the two steps; vii. PCC; viii. NaClO_2_, 81% over the two steps; ix. CH_2_N_2_; x. TBAF, 44% over the two steps, xi. LiOH, THF, MeOH, H_2_O, 45%.

**Scheme 20:**
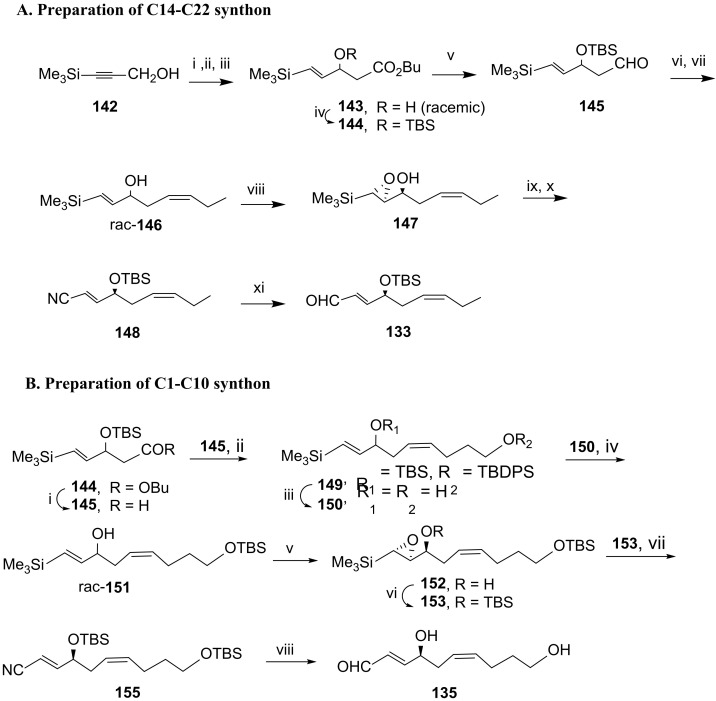
Preparation of C14-C22 synthon **133** (**A**) and C1-C10 synthon **135** (**B**). Reagents and conditions: (**A**). i. LiAlH_4_; ii. PCC; iii. MeCOOBu/LDA, 69% over the three steps; iv. TBSCl, imidazole, 89%; v. DIBAL; vi. PrPPh_3_Br, NaHMDS, THF, −90 °C to rt, 12 h, vii. TBAF, 74% over the three steps; viii. *t*BuOOH, Ti(OiPr)_4_, l-(+)-DIPT, CH_2_Cl_2_, −18 °C, 6 h, 47%, 98% ee; ix. TBSOTf, 2,6-lutidine, CH_2_Cl_2_, rt, 1.5 h; x. Et_2_AlCN, toluene, rt, overnight, 79% over the two steps; xi. DIBAL, CH_2_Cl_2_, −40 °C to 0 °C, 1 h, 73%. (**B**). i. DIBAL, −78 °C; ii. TBDPSO(CH_2_)_4_PPh_3_I, NaHMDS, THF, −90 °C to rt, 14 h; iii. TBAF, 91% from **144**; iv. TBSCl, CH_2_Cl_2_, 0 °C, 3.5 h, 84%; v. *t*BuOOH, l-(+)-DIPT, Ti(O*i*Pr)_4_, CH_2_Cl_2_, −18 °C, 6 h, 44% and 98% ee; vi. TBSOTf, 2,6-lutidine, CH_2_Cl_2_, rt, 3 h; vii. Et_2_AlCN, toluene, 0 °C, 2 h, 92% over the two steps; viii. DIBAL, CH_2_Cl_2_, −70 °C, 1 h, 82%.

In 2017, Kobayashi and co-workers [93] developed a new approach for the synthesis of RvD5 by assembling the key building blocks via Wittig reaction—namely, the C14-C22 aldehyde synthon **133**, which reacted with the C11-C13 phosphonium salt that, after deprotection of TBS, afforded the C11-C22 alcohol **136**, which was transformed in the iodide **137**. Reaction with triphenylphosphane afforded phosphonium salt **138**, the Wittig reaction of which with the C1-C10 aldehyde **135** gave RvD5 fully protected precursor **139** (Scheme 19). Selective TBS deprotection of the primary functionality with PPTS in methanol was followed by oxidation to carboxylic acid and esterification by reaction with diazomethane. Treatment with TBAF gave the ester **141** with the free hydroxy groups at positions 7 and 17, the hydrolysis of which with lithium hydroxide afforded RvD5 in 5.6% overall yield from aldehyde **133**. The synthesis of synthon **133** (Scheme 20A) started from commercially available 3-(trimethylsilyl) prop-2-yn-1-ol **142**. Reduction with lithium aluminium hydride to install the double bond, oxidation of the primary alcohol to aldehyde with pyridinium chlorochromate (PCC), and aldol reaction conducted to the preparation of ester **143** in 69% yield over the three steps. After protecting the hydroxy group with the *tert-*butyldimethylsilyl(TBS) group, DIBAL reduction generated the terminal aldehyde, the Wittig reaction of which with the ylide resulting from basic treatment of the triphenyl(propyl)phosphonium bromide, followed by TBS cleavage, gave racemic alcohol **146** in 74% yield over the three steps. Sharpless regio- and stereoselective epoxidation afforded epoxide **147** in 47% isolated yield. After protection of the hydroxy group with TBS, the nitrile was installed by reaction with diethylaluminum cyanide via epoxide ring opening and Peterson olefination to give **148**. Nitrile reduction with DIBAL afforded the aldehyde **133**, isolated in 73% yield.

The synthon **135** was prepared starting from compound **144** (Scheme 20B). Reduction with DIBAL to generate aldehyde **145** was followed by Wittig olefination, giving the *cis*-alkene **149**, desilylated with TBAF to afford diol **150** in 91% yield over the three steps. Regioselective protection of the primary alcohol with the TBS group was followed by Sharpless asymmetric epoxidation, giving compound **152** in high enantiomeric excess and 44% yield. Reaction with diethylaluminum cyanide afforded nitrile **155** in very high yield, which was finally reduced with DIBAL, giving the aldehyde synthon **135**, isolated in 82% yield.

**Figure 3:**
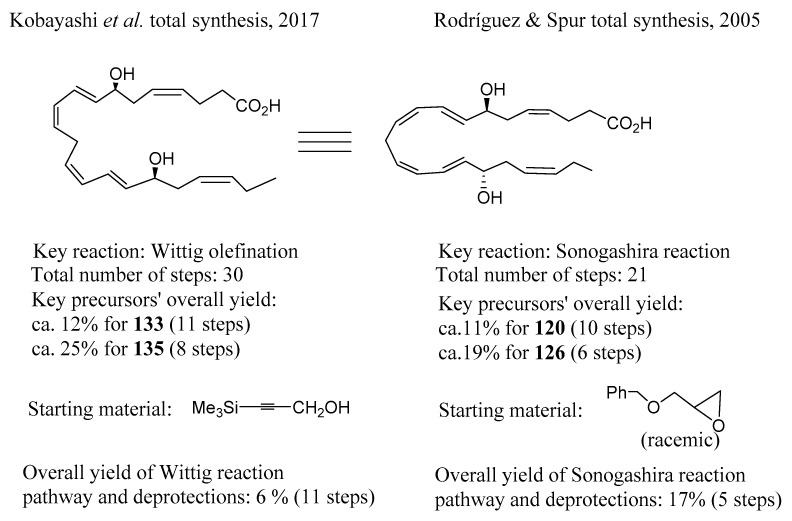
Summary of the total synthesis steps and overall yields of RvD5 [91,93].

In Figure 3, a summary of both the methodologies here reviewed for the total synthesis of RvD5 is given, indicating the number of steps required, the key reactions for assembling the building blocks, and the yields and number of steps required for the preparation of the building blocks. Both approaches are, indeed, strategic, very elegant, and successful, involving stereo- and regioselective reaction steps culminating in the total synthesis of RvD5, a complex structure with multiple *cis*- and *trans*-double bonds and two chirality centres with (*S*)-configuration.

#### 3.2.6. Resolvin D6

**Scheme 21:**
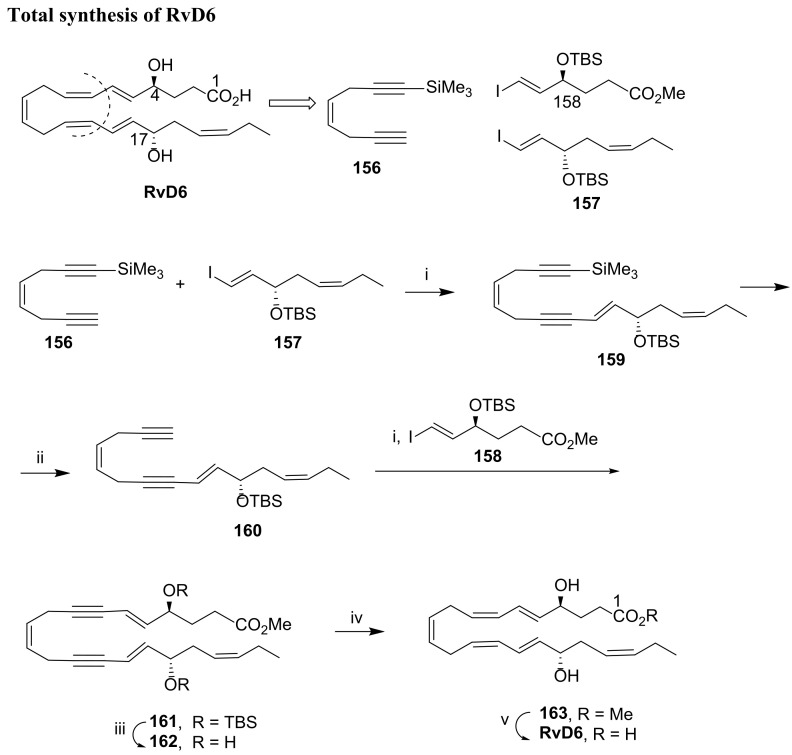
Total synthesis of RvD6, as reported by Rodríguez and Spur [94]. Reagents and conditions: i. Pd(PPh_3_)_4_, CuI, PrNH_2_, benzene, rt; ii. AgNO_3_, CH_3_OH, H_2_O, NaCN, 0 °C; iii. MeCOCl, CH_3_OH, 0 °C to rt; iv. Zn(Cu/Ag), CH_3_OH, H_2_O, 50 °C; v. 1 N LiOH, H_2_O, THF, 0 °C, then H^+^ (NaH_2_PO_4_ saturated) (yields were not provided).

In 2012, the first total synthesis of RvD6 was reported by Rodríguez and Spur [94]. Sonogashira coupling was the key reaction to assemble the core structure of RvD6, and the key precursors are compounds **156**, furnishing the triple bond and iododerivatives **157** and **158** (Scheme 21). The first Sonogashira reaction coupled **156** with the iodide **157** under Pd(0)/CuI catalysis to give the intermediate **159**. Cleavage of the trimethylsilyl group by treatment with sodium nitrate, followed by sodium cyanide work-up gave alkyne **160**, which was coupled with the iodoester **158** through the second Sonogashira reaction. After TBS deprotection, Zn(Cu/Ag) reduction of **162** afforded **163** embodying the core structure of RvD6, which was obtained after mild alkaline ester hydrolysis.

**Scheme 22:**
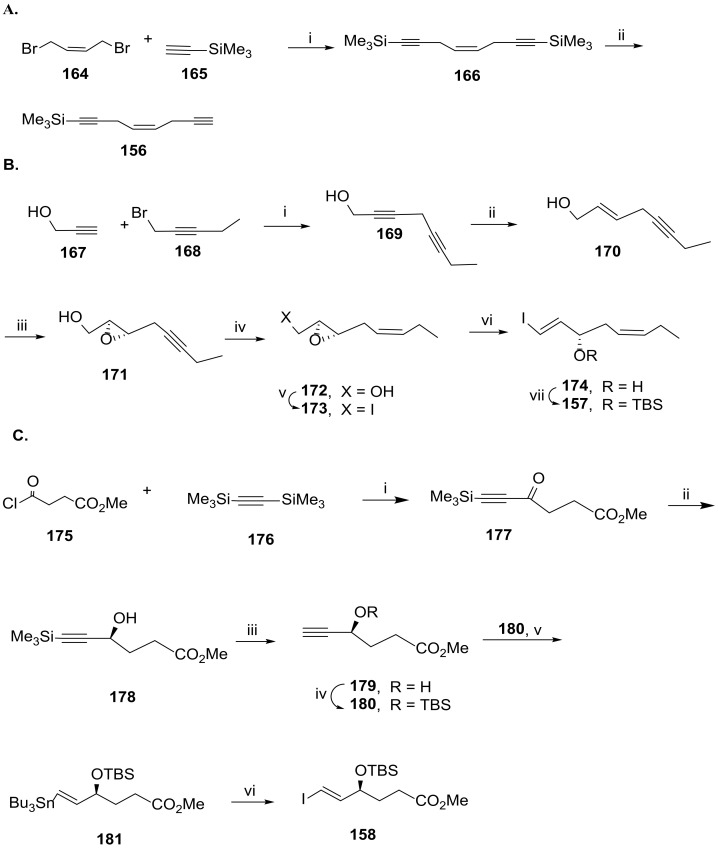
Synthesis of Sonogashira coupling partners, as reported by Rodríguez and Spur [94]. (**A**). i. CuI, Na_2_SO_3_, K_2_CO_3_, DMF, rt; ii. AgNO_3_, CH_3_OH, H_2_O, NaCN, 0 °C; (**B**). i. DBU, CuI, HMPA, THF; ii. LiAlH_4_, ether, 0 °C to rt; iii. l-(+)-DMT, Ti(O*i*Pr)_4_, TBHP, molecular sieves 4 Å, CH_2_Cl_2_; iv. H_2_, Lindlar cat., Et_3_N, hexane; v. I_2_, Ph_3_P, imidazole, *i*Pr_2_EtN, CH_3_CN, ether; vi. NaHMDS, DMF, −60 °C; vii. TBSCl, imidazole, DMF, 67%; (**C**). i. 10% InBr_3_, CH_2_Cl_2_, 0 °C to rt, 94%; ii. Ru[(*S*,*S*)-TsDPEN](*p*-cymene), *i*PrOH, rt; iii. KF·2H_2_O, 18-crown-6, DMF, rt; iv. TBSCl, imidazole, DMF, 0 °C to rt, 63% over the three steps; v. Bu_3_SnH, AIBN, 130 °C; vi. I_2_, ether, 0 °C, 93%.

As the key precursors are not commercially available, their preparation has been accomplished. The key precursor **156** was synthesized starting from 1,4-dibromobut-2-ene (**164**) in two steps (Scheme 22A). Treatment with trimethylsilylacetylene excess (**165**) in DMF at room temperature gave **166** in 34% isolated yield, which was desilylated with silver nitrate (2 equiv) and work-up with sodium cyanide, providing **156** by extraction with hexane/ethyl acetate. The configuration of the chirality centre at RvD6 C17 was introduced in precursor **157** via Sharpless asymmetric epoxidation (Scheme 22B). Its synthesis started with the reaction of propargyl alcohol **167** with 1-bromopent-2-yne (**168**) to provide octa-2,5-diyn-1-ol (**169**). Triple bond reduction with lithium aluminum hydride was regioselective affording the enynol **170** in 70% yield. Stereoselective Sharpless epoxidation gave **171**, and Lindlar reduction of the triple bond installed the *cis*-double bond present in **172**. Replacement of the hydroxy group by iodide gave epoxide **173**, which was transformed into the alcohol **174** with the required configuration by elimination with NaHDMS and concomitant epoxide ring opening. Hydroxy group protection with TBS provided the key precursor **157** in 67% yield.

The preparation of **158** was accomplished starting with reaction of methyl 4-chloro-4-oxobutanoate (**175**) with bis(trimethylsilyl)acetylene catalysed by indium bromide, affording the alkinyl derivative **177** in 94% yield (Scheme 22C). The key reaction to install the configuration of the chirality centre at RvD6 C4 is the asymmetric transfer hydrogenation, producing the chiral intermediate **178** with more than 95% enantiomeric excess. After desilylation with potassium fluoride in the presence of catalytic crown ether and TBS protection of the hydroxy group to give **180**, free radical addition with excess tributyltin hydride in the presence of catalytic amount of azobisisobutyronitrile gave **181** in moderate yield. Reaction with iodine afforded the key precursor **158** in 93% yield.

**Scheme 23:**
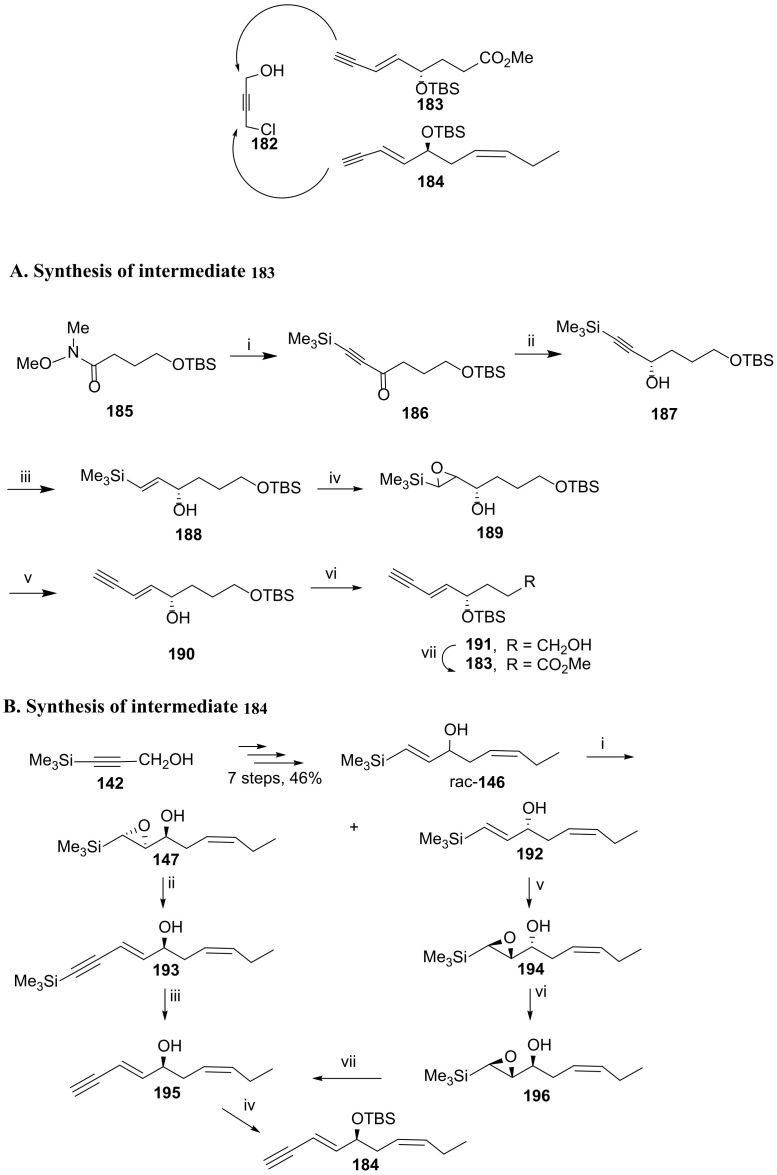
Strategy for the synthesis of RvD6 and preparation of the intermediates **183** and **184**, as reported by Kobayashi and co-workers [95]. Reagents and conditions: (**A**). i.Me_3_Si-C≡C-Li (1.7 equiv.), THF, −78 °C to 0 °C, 99%; ii. Ru[(*S*,*S*)-TsDPEN](*p*-cymene), *i*PrOH, rt, 1 h, 96% yield and 98% ee; iii. Red-Al, Et_2_O, rt, 4 h, 59%; iv. *m*-CPBA, NaHCO_3_, rt, 71%, *syn*/*anti* 3:2; v. 1. Me_3_Si-C≡C-Li (8 equiv.), THF, HMPA, rt, 5 h; 2. K_2_CO_3_ (5 equiv.), MeOH, rt, 2 h, 42% (2 steps); vi. 1. TBSCl, imidazole, 92%; 2. PPTS (cat.), CH_2_Cl_2_, MeOH, rt, 4 h, 55% [recovered bis (TBS ether), 33%]; vii. 1. (COCl)_2_, DMSO, Et_3_N; 2. NaClO_2_, Me_2_C = CHMe; 3. CH_2_N_2_, 51% from **191**. (**B**). i. *t*BuOOH, Ti(O*i*Pr)_4_, l-(+)-DIPT, 47% (**147**, 99% ee) and 42% (**192**, 99% ee); ii. Me_3_Si-C≡C-Li (6 equiv.), THF, HMPA, rt, 6 h; iii. K_2_CO_3_ (1.2 equiv.), MeOH, rt, 3 h, 78% (over two steps); iv. TBSCl, imidazole, 89%; v. *t*BuOOH, Ti (O*i*Pr)_4_, d-(−)-DIPT, CH_2_Cl_2_, −18 °C, 6 h, 89%; vi. 1. 4-(NO_2_) C_6_H_4_CO_2_H, PPh_3_, DIAD; 2.2N NaOH aq., 85%; vii. 1. Me_3_Si-C≡C-Li (4 equiv.), THF, HMPA, rt, 4 h; 2. K_2_CO_3_, MeOH, 78% from **196**.

**Scheme 24:**
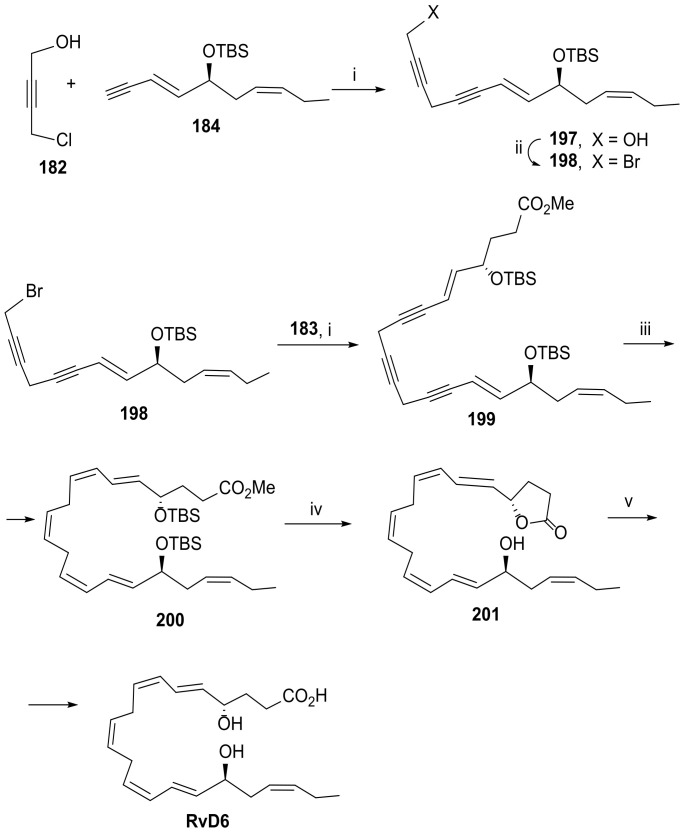
Synthesis of RvD6, as reported by Kobayashi and co-workers [95]. Reagents and conditions: i. NaI (2 equiv.), CuI (2 equiv.), CsCO_3_ (1.4 equiv.), DMF, rt, 22h for **197** and 7 h for **199**, 82%; ii. CBr_4_, PPh_3_, 73% for **198** over the two steps; iii. Zn(Cu/Ag) (1600 equiv.), MeOH/H_2_O, 40 °C, 37 h, 76%; iv. TBAF (8 equiv.), THF, rt, 1 h, 53%; v. LiOH·H_2_O (2 equiv.), MeOH/THF/H_2_O, rt, 1 h, 79%.

Kobayashi and co-workers [95] have recently reported another approach for the total synthesis of RvD6, by using intermediates **182**, **183**, and **184** (Scheme 23), envisioning a strategy based on Sonogashira coupling of **183** and **184** to **182**, followed by hydrogenation with experimental conditions, affording the required double bonds (Scheme 24).

The preparation of **183** was accomplished starting from amide **185**, synthesized from butyrolactone [96]. After reaction with trimethylsilylacetylide for the introduction of the triple bond and stereoselective reduction of the carbonyl group with the Noyori catalyst [97], reduction with Red-Al provided the hex-1-en-3-ol derivative **188**. Epoxidation was followed by reaction with the lithium trimethylsilylacetylide and trimethylsilyl desilylation to afford alcohol **190** containing the (*E*)-enyne moiety. Protection of the hydroxy group with TBSCl and regioselective deprotection of the primary position gave **191**. Intermediate **183** was achieved by Swern oxidation of **191** to carboxylic acid and esterification. The overall yield for the formation of this intermediate was 4.3% from amide **185**.

The synthesis of intermediate **184** started from the racemic allylic alcohol **146** (Scheme 23B), obtained in 46% yield overall yield (seven steps) from 3-trimethylsilylprop-2-yn-1-ol and already applied for the preparation of RvD5 (Scheme 20). Asymmetric epoxidation gave epoxide **147** and the allylic alcohol **192**, both used for the synthesis of **184**. The epoxide reacted with lithium trimethylsilylacetylide to afford alcohol **193**, which was desilylated and then protected with the group TBS. This synthetic pathway provided **184** with the overall yield of 15% from 3-trimethylsilylprop-2-yn-1-ol (**142**), involving eleven steps. The alternative pathway started from allylic alcohol **192**, which was converted to **194**, the enantioner of **147**, by Sharpless asymmetric epoxidation. Inversion of the configuration of the centre of chirality linked to the hydroxy group was achieved by Mitsunobu reaction, affording **196** in high yield. Reaction with lithium acetylide at room temperature was completed in four hours, giving alcohol **195**, the hydroxy group of which was then protected with TBS by reaction with TBSCl in the presence of imidazole. The overall yield of this route was 10%, covering twelve steps. Sonogashira coupling was a key reaction to access RvD6 core structure. The coupling of chloropropynol **182** and the triple bond containing partner **184** afforded **197**, which was transformed in the bromoester **198** by treatment with tetrabromomethane and triphenylphosphane. A second Sonogashira coupling of **198** with reaction partner **183** gave precursor **199** in 82% yield. The next key reaction is the reduction of the triple bonds, achieved with Zn(Ag/Cu) in high excess (1600 equivalent) for the reduction of the non-conjugated triple bond, installing RvD6 core structure in compound **200**. Treatment with *tert*-butylammonium fluoride led to desilylation and to an intramolecular cyclization with the formation of a butyrolactone, the hydrolysis of which with lithium hydroxide hydrate afforded RvD6 (Scheme 24), in an overall yield of 19% from **182**.

## 4. Protectins from DHA

DHA is also the precursor for the biosynthesis of protectins, also called neuroprotectins when they are produced in neural systems [98]. The “neuro” prefix before protectin D1 (NPD1) results from the biosynthesis location and the potent neuroprotective effects identified [98]—namely, protective actions in the retina and brain and in the induction of pain [99,100,101]. The anti-inflammatory and pro-resolving protectins have EC_50_ values in the low nanomolar to picomolar range [77], also exerting anti-apoptotic activity [102]. Protectins beneficial effects against obesity and diabetes have been investigated. Gonzales-Periz et al. [103] reported that biosynthetic formation of SPMs was deregulated in obese mice and in inflamed white adipose tissues, and reduced insulin resistance was observed in white adipose tissues, with DP1 and RvD1 being the dominant DHA-derived SPMs, based on LC/MS-MS. Additionally, Clària et al. [104] showed that an unbalanced level of SPMs are directly connected to insufficient tissue resolution in both in vitro and in vivo models of diabetes and obesity. As the chronic unresolved inflammation in adipose tissues may result in insulin resistance, diabetes, and fatty liver disease, endogenous SPMs such as the PDs, displaying potent pro-resolving and anti-inflammatory bioactions in vivo, become important biotemplates towards drug development targeting obesity and diabetes [77].

### 4.1. Biosynthesis of Protectin D1

**Scheme 25:**
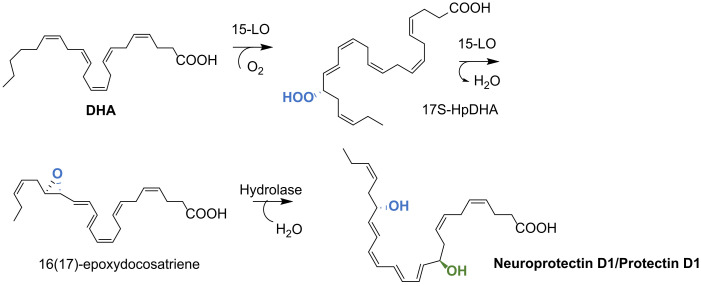
NPD1/PD1 biosynthesis.

The biosynthesis of NPD1/PD1 is initiated with the formation of 17*S*-HpDHA by 15-LO, leading to the 16(17)-epoxydocosatriene intermediate, which is converted by a hydrolase to the final product PD1/NPD1 with the correct double bond geometry [26] (Scheme 25).

### 4.2. Total Synthesis of Protectin D1

The total synthesis of stereochemically pure NPD1/PD1 has been accomplished by several approaches, as disclosed by Ogawa and Kobayashi in 2011 [105], by Serhan and co-workers in 2012 [106], by Rodríguez and Spur in 2014 [107], and more recently by Sala and co-workers in 2019 [105].

#### 4.2.1. Ogawa and Kobayashi Approach

**Scheme 26:**
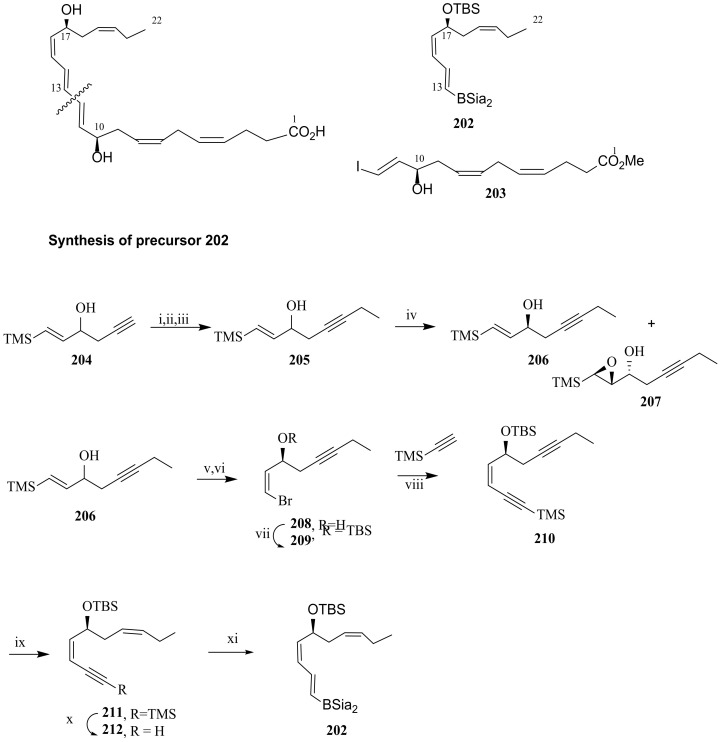
Synthesis of precursor **202**, as reported by Ogawa and Kobayashi [105]. Reagents and conditions: i. CH_2_=CHOEt PPTS; ii. BuLi then EtBr, DMPU; iii. 3 N HCl, 71% over the three steps; iv. *t*BuOOH D-DIPT, Ti(OPr)_4_, –20 °C, 12 h, 48% for **206**, 99% ee and 46% for **207**, 99% ee; v. Br_2_; vi. TBAF, vii. TBSCl, 65% over the three steps; viii. PrNH_2_, CuI, Pd (PPh_3_)_4_, 89%; ix. H_2_, Pd/BaSO_4_, EtOAc, 94%; x. K_2_CO_3_, MeOH, 90%; xi. Sia_2_BH (1.5 equiv.), 0 °C, 1 h, THF.

In 2011, a stereoselective total synthesis was reported by Ogawa and Kobayashi [105], the strategy of which was based on the Suzuki coupling to construct PD1 core structure, by reacting C22-C13 vinylborane **202** with the vinyl iodide **203** (Scheme 26). The preparation of **202** started by alkylation of **204** with ethyl bromide to give racemic **205**, further subjected to the asymmetric Sharpless epoxidation, which gave enantiomerically pure alcohol **206** with the required (*S*)-configuration and the epoxide **207** in 48% and 46% yield, respectively. Reaction of **206** with bromine and tetrabutylammonium fluoride gave the bromoalcohol **208**, which was protected with TBS to afford **209** in good yield (Scheme 26). Sonogashira coupling with trimethylsilyl acetylene proceeded in very high yield, giving **210**, the regio- and stereoselective hydrogenation of which provided compound **211**, which was desilylated with potassium carbonate prior to reaction with bis(siamyl)borane to give **202**.

**Scheme 27:**
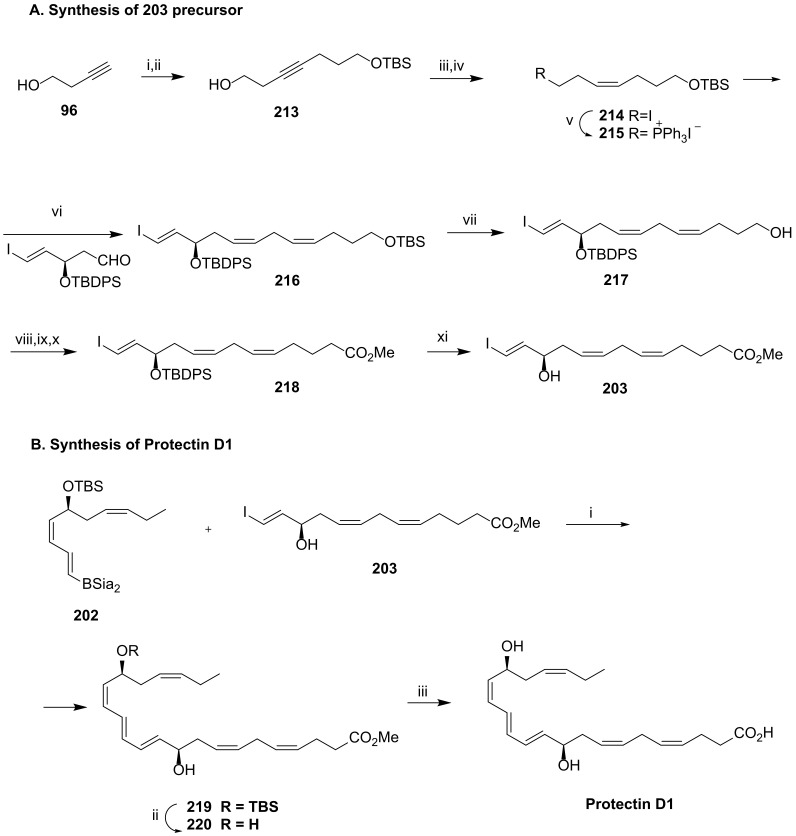
Synthesis of precursor **203** (**A**) and of PD1 (**B**), as reported by Ogawa and Kobayashi [105]. Reagents and conditions: (**A**). i. LiNH_2_, NH_3_/THF; ii. Br (CH_2_)_3_OTBS, 81%; iii.H_2_,Lindlar cat., quinoline; iv. I_2_, PPh_3_, imidazole; v. PPh_3_, MeCN, 71% over the last three steps; vi. NaHMDS, 94%; vii. PPTS, CH_2_Cl_2_/EtOH (1:1), quant.; viii. SO_3_•Py; ix. NaClO_2_; x. CH_2_N_2_, 73%; xi. TBAF, 90%. (**B**). i. Pd (PPh_3_)_4_ (20 mol%) 2 N NaOH (10 equiv.) THF, rt, 1 h; ii. TBAF; iii. 2 N L iOH THF, H_2_O, 42%.

The synthesis of **203** started with the chain elongation of **96**, giving **213** in 81% yield (Scheme 27A). Hydrogenation of the triple bond to the *cis* double bond succeeded with the Lindlar catalyst in ethyl acetate, in the presence of quinoline to avoid over reduction. Treatment of **213** with iodine and triphenylphosphane generated the iodo derivative **214**, which was transformed in the phosphonium salt **215** by further addition of triphenylphosphane. A stereoselective Wittig reaction of the phosphorane, obtained by addition of NaHMDS to the phosphonium salt **215**, with the aldehyde afforded the iodotriene **216**, partially desilylated at the primary position by the addition of PPTS. Oxidation of the primary alcohol to carboxylic acid and esterification with diazomethane gave the ester **218**, desilylated to afford **203**, synthesized from **96** in 37.8% overall yield.

PD1 synthesis was then achieved by Suzuki coupling of **202** and **203** (Scheme 27B), followed by desilylation with TBAF and ester hydrolysis with aq. lithium hydroxide in 42% yield.

#### 4.2.2. Serhan Approach

**Scheme 28:**
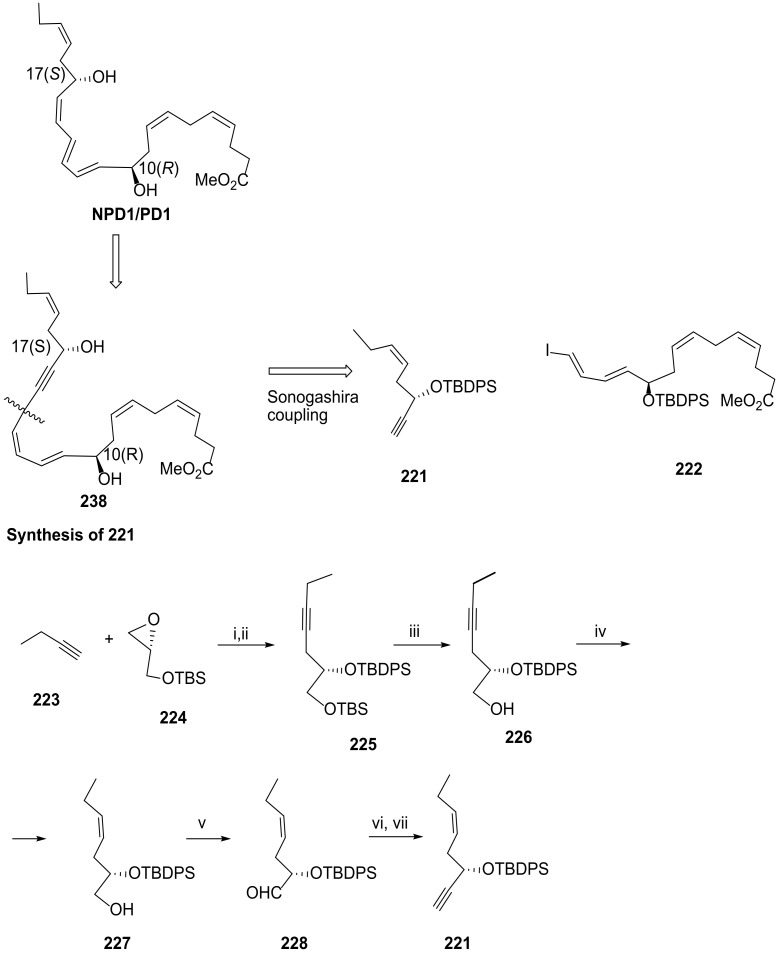
Sonogashira reaction partners for the total synthesis of PD1 and preparation of **221**, as reported by Serhan and co-workers [106]. Reagents and conditions: i. *n*BuLi, BF_·_OEt_2_, −78 °C, THF, 84%; ii. TBDPS–Cl, imidazole, DMAP, rt, CH_2_Cl_2_, 89%; iii. CSA, rt, CH_2_Cl_2_/MeOH, 78%; iv. H_2_/Lindlar cat., quinoline, rt, EtOAc, 95%; v. DMSO, (COCl)_2_ Et_3_N, −78 °C, CH_2_Cl_2_, 98%; vi. PPh_3_, CBr_4_, 0 °C, CH_2_Cl_2_,72%; vii. *n*BuLi, Et_2_O, 83%.

**Scheme 29:**
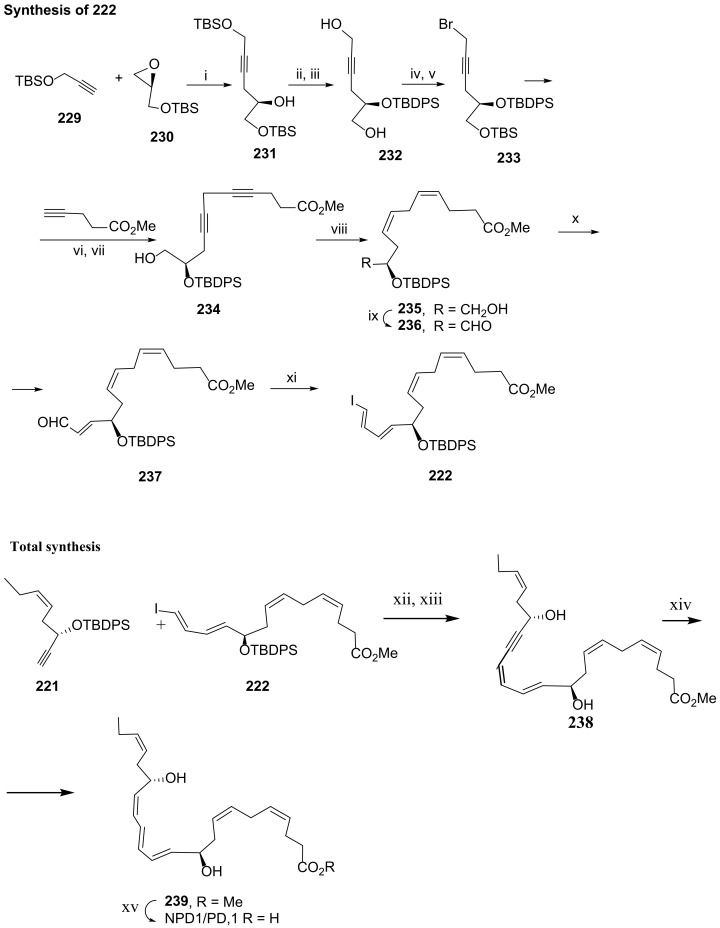
Preparation of **222** and total synthesis of NPD1/PD1, as reported by Serhan and co-workers [106]. Reagents and conditions: i. *n*BuLi, BF_3_·Et_2_O, −78 °C, THF, 77%; ii. TBDPS–Cl, imid., DMAP, rt, CH_2_Cl_2_, 97%; iii. CSA, rt, CH_2_Cl_2_/MeOH, 81%; iv.NBS, PPh_3_, 0 °C, CH_2_Cl_2_, 80%; v. TBSOTf, lutidine, 0 °C, CH_2_Cl_2_, 89%; vi. CuI, NaI, K_2_CO_3_, rt, DMF, 75%; vii. CSA, rt, CH_2_Cl_2_/MeOH, 88%; viii. H_2_/Lindlar cat., quinoline, rt, EtOAc, 68%; ix. DMSO, (COCl)_2_, Et_3_N, −78 °C, CH_2_Cl_2_, 98%; x. Ph_3_P=CHCHO, PhMe, reflux, 2 h, 90%; xi. CHI_3_, CrCl_2_, 0 °C, THF, 67%; xii. Pd(PPh_3_)_4_ (cat.), CuI, rt, PhH, 96%; xiii. TBAF, THF, rt, 2 h, 65%; xiv. Zn(Cu/Ag), MeOH/H_2_O, 40 °C, 60%; xv. NaOH, MeOH/H_2_O, rt, 95%.

**Scheme 30:**
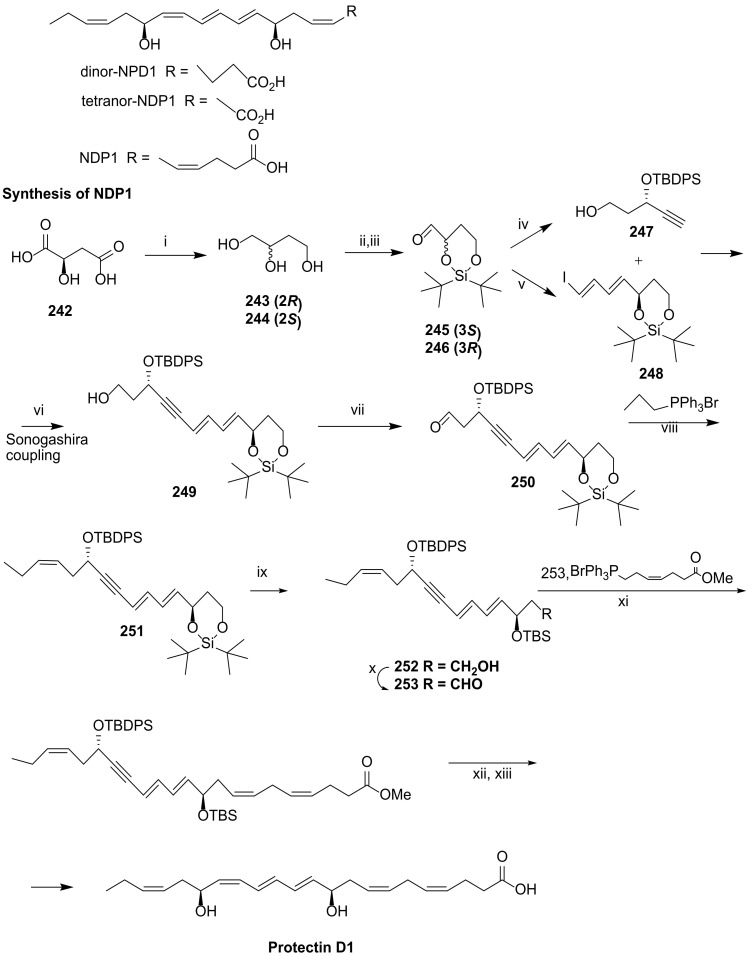
Synthesis of protectin D1/NPD1, as reported by Balas and co-workers [108]. Reagents and conditions: i. BH_3_.SMe_2,_ B(OMe)_3_,98%; ii. *t*Bu_2_Si(OTf)_2_, py, CH_2_Cl_2_/DMF (6:4), −30 °C, 2 h; iii. 1. (COCl)_2_, DMSO; 2. Et_3_N, CH_2_Cl_2_, −78 °C to rt, 1 h, 90% over the two steps; iv. 1. (MeO)_2_P(=O)-C(=N_2_)-P(=O)-CH_3_, K_2_CO_3_, MeOH, 0 °C to rt, 3 h; 2. TBAF, THF, 0 °C to rt, 2 h; 4. 1. TESCl, Et_3_N, CH_2_Cl_2_, −25 °C, 15 h; 5. TBDPSCl, TES deprotection, 44% over the 5 steps; v. 1. Ph_3_P=CHCHO, CH_3_CN, 30 °C; Me_3_SiCHN_2_, LDA, −78 °C to 0 °C, THF, 2.5 h; 3. a. ZrCl_2_(Cp)_2_, DIBAL, b. THF, I_2_; 52% over the three steps; vi. Pd (PPh_3_)_4_, CuI, THF, 91%; vii. 1. (COCl)_2_, DMSO; 2. Et_3_N, CH_2_Cl_2_, −78 °C to rt, 1 h,95%; viii. NaHMDS, THF, −78 °C, 3 h; ix. 1. TBAF, 0 °C to rt, 1 h; 2. TBSCl, imidazole, CH_2_Cl_2_, 0 °C to rt, 1 h; 3. PPTS, EtOH, 0 °C to rt, 5.5 h; x. 1. (COCl)_2_, DMSO 2. Et_3_N, CH_2_Cl_2_, −78 °C to rt, 1 h, 33% over the last five steps; xi Base, THF; xii. Zn(Cu-Ag),26%; xiii. LiOH (1M), MeOH, 97%.

Serhan and co-workers [106] reported, in 2012, a highly stereocontrolled synthesis of NPD1/PD1 based on a Sonogashira coupling of reaction partners **221** (Scheme 28) and **222** (Scheme 29). The 10(*R*) and 17(*S*) configurations were established through ring opening of enantiomerically pure epoxides **224** for the configuration 17(*S*) (Scheme 29) and **230** for the configuration 10(*R*) (Scheme 30). (*Z*)-alkenes were obtained by hydrogenation of the alkynes with the Lindlar catalyst in ethyl acetate at room temperature, in the presence of quinoline, obtaining **227** in very high yield (95%) (Scheme 29) and **235** in 68% yield (Scheme 30). In addition, the triene (11*E*,13*E*,15*Z)* was introduced at the very end of the synthetic pathway, by triple bond reduction after the Sonogashira reaction of **221** with **222** to avoid *Z*/*E* isomerization. The synthesis of **221** was accomplished by Swern oxidation of the primary alcohol of **227** to afford aldehyde **228**, submitted then to the Corey–Fuchs reaction [105] (Scheme 29), giving **221** obtained in 32.4% overall yield, covering the required protection and deprotection steps involved in this synthetic route.

The synthesis of **222** (Scheme 30) succeeded by Swern oxidation of **235** to aldehyde **236**, followed by Wittig reaction to install the *α,β*-unsaturated aldehyde, which was subjected to Takai olefination to afford **222**, synthesized in 11.4% overall yield. The total synthesis ended with the Sonogashira reaction coupling **221** and **222** in 96% yield, desilylation, Zn(Ag/Cu) reduction of the triple bond, and ester hydrolysis (Scheme 30).

#### 4.2.3. Approach by Balas and Sala

In 2019, Balas and Sala groups [108] investigated the metabolic process of NPD1 and reported a new total synthesis of NPD1 and that of its two major metabolites, tetranor-NDP1 and dinor-NDP1 (Scheme 30), giving the first evidence of β-oxidation in SPMs. Noteworthily, tetranor-NDP1 kept the bioactivity of NPD1 by inhibiting neutrophil chemotaxis in vitro and neutrophil tissue infiltration in vivo, while dinor-NDP1 was not active. The key reaction for building the NDP1 structure core is the Sonogashira coupling of **245** and **246** for the preparation of the key intermediate **247** (Scheme 30). The installation of the two chirality centres with the required configuration was achieved by using (2S)-butane-1,2,4-triol (**241**) as starting material for the preparation of (**245**) and its enantiomer **242** for the synthesis of **246**, both accessed in five reaction steps. Although the (*R*)-triol enantiomer is commercially available, the synthetic route became cheaper when it was prepared starting from malic acid with the (*R*) configuration. Silyl protection of the 2,4-diol and Swern oxidation of the remaining primary alcohol gave compounds **243** and **244** in high yield. The synthesis of pent-4-yn-ol 245 was achieved with by reaction of the aldehyde **243** with dimethyl(1-diazo-2-oxopropyl)phosphonate, the Bestmann–Ohira reagent to prepare alkynes from aldehydes [109], followed by a sequence of protection and deprotection steps which furnished **245** in 44% yield from **243**. The iododiene **246** was achieved by a three reaction pathway starting with a Wittig reaction to furnish an α,β-unsaturated aldehyde which underwent a Colvin rearrangement with trimethylsilyldiazomethane and lithium diisopropylamide, affording a terminal alkyne, the quantitative hydrozirconation of which, followed by iodination [108,110], furnished **246** in 52% yield from **244**. Swern oxidation of Sonogashira reaction product **247** furnished aldehyde **248**, the Wittig reaction of which with the phosphorane generated by triphenyl(propyl)phosphonium bromide and sodium hexamethyldisilazane (NaHDMS) afforded **249**, embodying the terminal *(Z)*-alkene as required. Desilylation with TBAF, protection with TBSCl, and deprotection of the primary alcohol with PPTS gave **250**, which underwent Swern oxidation to aldehyde **251**. A Wittig reaction was used to introduce Protectin D1 fragment C1-C8, followed by TBDPS deprotection with TBAF, triple bond reduction with Zn(Ag-Cu) in 26% yield over the three steps. Ester hydrolysis succeeded with aqueous LiOH to give NDP1 in 97% yield. This synthetic pathway covered 27 steps, furnishing NPD1 in 2.84% yield.

## 5. Maresins from DHA

Maresins have been discovered more recently, being the third-largest family of SPMs which are biosynthesized from DHA. Biosynthesis of maresins starts in M2 macrophages and is initiated by an epoxygenation reaction (see Section 5.1). Maresin 1 (MaR1) and maresin 2 (MaR2) are active anti-inflammatory mediators with a powerful protective effect in inflammation, oxidative stress, and immune diseases as protective mediators of macrophage function [85]. Maresins protect the human body, limiting neutrophil infiltration, enhancing macrophage phagocytosis, reducing the production of pro-inflammatory factors, stimulating tissue regeneration, and controlling pain. MaRs derived from DHA show action in metabolic diseases, nervous system diseases, and kidney and inflammatory bowel diseases, as well as in tissue regeneration and pain control [77,86]. MaR-1 appears to be a molecule with potential for the treatment of motor neuron diseases such as amyotrophic lateral sclerosis (ALS) or spinal muscular atrophy (SMA), which are fatal neurodegenerative diseases that cause loss of motor function and progressive degeneration [55]. In addition, MaR1 attenuates the generation of reactive oxygen species in response to high glucose levels [87], reduces kidney injury and serum creatinine levels in a sepsis-associated kidney injury model [81], and preserves kidney function and inhibits NF-κB activity in a renal ischaemia–reperfusion injury model [88].

### 5.1. Biosynthesis of Maresin 1 and Maresin 2

**Scheme 31:**
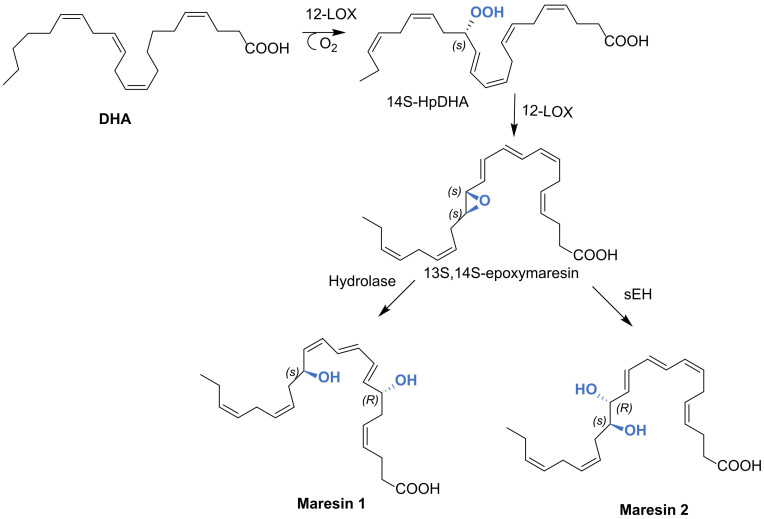
Maresin 1 and maresin 2 biosynthesis [104,111].

The biosynthesis of maresins occurs primarily in M2 macrophages. The first reaction involves the formation of a hydroperoxyDHA on the fourteenth carbon atom by 12-lipoxygenase, a key enzyme in the synthesis of maresins, generating 14*S*-HpDHA. This molecule suffers epoxidation through 12-LOX being converted to 13*S*,14*S*-epoxy-maresin. MaR-1 and Mar-2 are produced by enzymatic hydrolysis with epoxide hydrolase and with soluble epoxide hydrolase (sHE), respectively, as depicted in Scheme 31. [25,40,77,85,86,89,90].

### 5.2. Total Synthesis of Maresin 1 and Maresin 2

**Scheme 32:**
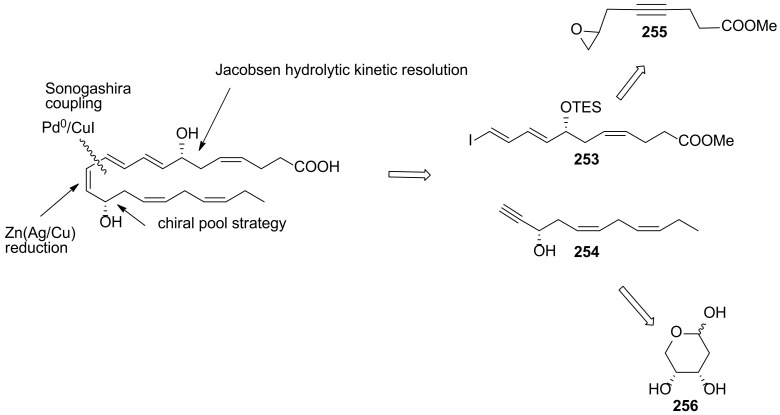
Retrosynthesis of maresin 1, as reported by Rodríguez and Spur [113].

The first total synthesis of maresin 1 was presented in 2011 by Inoue et al. [112], although it was not assigned to maresin 1 because the stereochemistry of the C7 chirality centre was unknown, and the total synthesis of maresin 1 with the established structure given in Scheme 32 was reported in 2012 by Rodríguez and Spur [113]. Their synthetic strategy was based on a Sonogashira reaction coupling compounds **253** and **254**. Scheme 32 illustrates the retrosynthesis of Mar1, showing that the triple bond resulting from Sonogashira coupling is reduced to a *cis* double bond with Zn(Ag/Cu), while the configuration of the C14 chirality centre is introduced by Jacobsen kinetic resolution of epoxide racemic mixture **255**. The C12-C22 fragment was prepared from 2-deoxy-d-*erythro*-pentopyranose **256**, used as the source of chirality for C17.

**Scheme 33:**
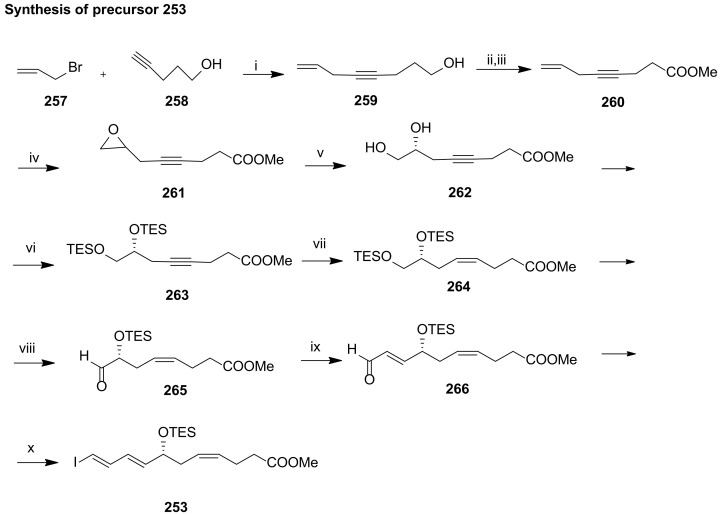
Synthesis of precursor **253**, as reported by Rodríguez and Spur [113]. Reagents and conditions: i. Na_2_SO_3_, cat.CuI, K_2_CO_3_, H_2_O, 92%; ii. Jones reagent, acetone, 69%; iii.10%Me_3_SiCl, CH_3_OH, 2,2-dimethoxypropane, rt, 90%; iv. *m*CPBA, NaHCO_3_, CH_2_Cl_2_, 0 °C, 75%; v. (*S*,*S*)-(salen)Co (III)(OAc)catalyst, H_2_O, ether, 0 °C to rt, 45%; vi. TESCl, imidazole, Et_3_N, DMF, 0 °C to rt, 95%; vii. H_2_, Lindlar cat., hexane, 99%; viii. (COCl)_2_, DMSO, CH_2_Cl_2,_ then Et_3_N, 83%; ix. Ph_3_P=CH–CHO, benzene, 70 °C, 43%; x. CrCl_2_, CHI_3_, THF, 0 °C, 69%.

**Scheme 34:**
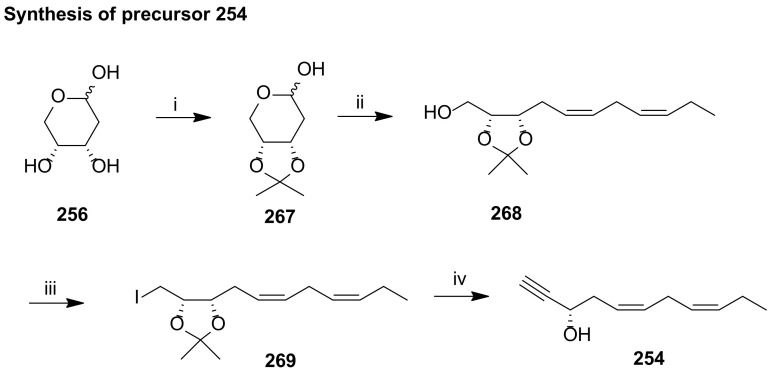
Synthesis of precursor **254**, as reported by Rodríguez and Spur [113]. Reagents and conditions: i.2-methoxypropene, PPTS, EtOAc, 0 °C to rt, 50%; ii. (*Z*)-CH_3_CH_2_CH=CH(CH_2_)_2_PPh_3_I^−^, BuLi, THF, −78 °C to 0 °C, 81%; iii. I_2_, Ph_3_P, imidazole, toluene, 60 °C,82%; iv. LDA, THF, −78 °C, 53%.

**Scheme 35:**
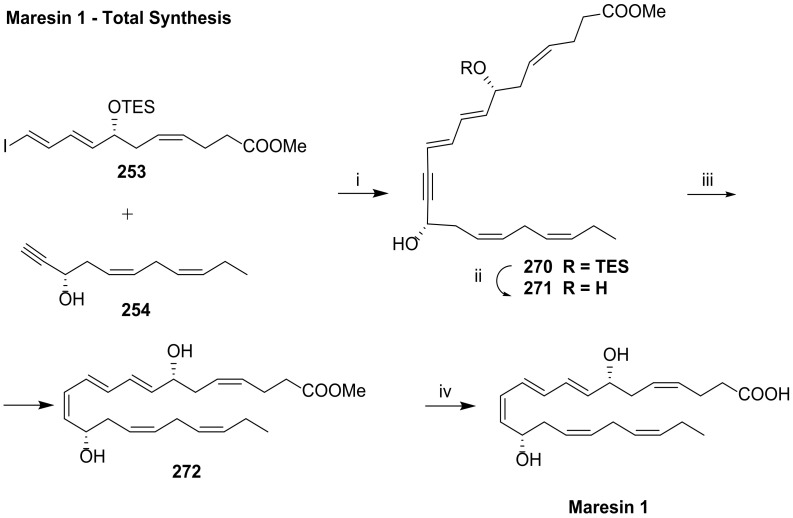
Synthesis of maresin 1, as reported by Rodríguez and Spur [113]. Reagents and conditions: i. Pd(PPh_3_)_4_, CuI, piperidine, benzene, rt, 81%; ii. PPTS, CH_3_OH, 0 °C,96%; iii. Zn(Cu/Ag), CH_3_OH, H_2_O, 40–45 °C, 66%; iv. 1N LiOH, CH_3_OH, H_2_O, 0 °C, then satd. NaH_2_PO_4_, 77%.

The preparation of the C1-C11 fragment **253** was achieved in 10 steps in 4.5% overall yield, starting with the coupling of allyl bromide **257** with pent-4-yn-1-ol **258** catalysed by CuI, followed by Jones oxidation and esterification (Scheme 33). The epoxidation of the terminal double bond with *m*-chloroperoxybenzoic acid gave racemic epoxy ester **261**. Jacobsen hydrolytic kinetic resolution of 261 with H_2_O and 5% (*S*, *S*)-salen-Co catalyst afforded **262** with more than 95% ee. Diol protection with triethylsilyl chloride and triple bond reduction with Zn(Ag/Cu) furnished **264**, which was regioselectively deprotected at the primary position to be subjected to Swern oxidation to aldehyde **265**. Wittig reaction furnished the α,β-unsaturated aldehyde **266**, converted to **253** by Takai olefination [92]. The Sonogashira reaction partner **254** was prepared in only four steps starting from **256**, which was protected with isopropylidene and subjected to Wittig reaction, furnishing **268** in good yield (Scheme 34). Iodination and LDA induced deprotonation–elimination gave the target compound **254** in 17.6% overall yield. The total synthesis of maresin 1 succeeded by Sonogashira coupling of **253** and **254** in good yield (Scheme 35). TES deprotection with PPTS, Lindlar reduction of the triple bond and ester hydrolysis gave maresin 1 in high yield, which co-eluted with a sample of natural maresin 1.

**Scheme 36:**
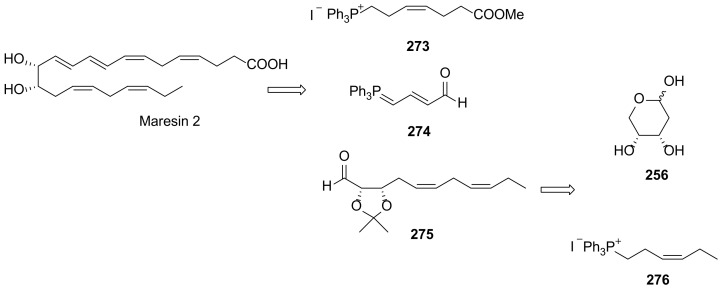
Retrosynthetic approach of maresin 2, as reported by Rodríguez and Spur [64].

**Scheme 37:**
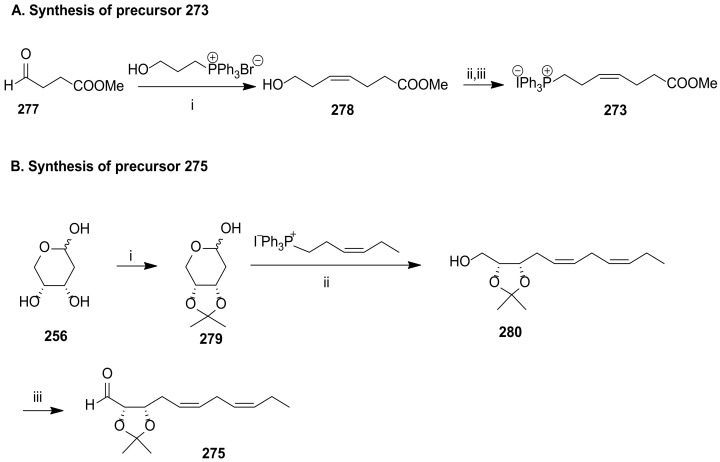
Synthesis of precursors **273** (**A**) and **275** (**B**), as reported by Rodríguez and Spur [64] and by Balas and co-workers [110]. Reagents and conditions: (**A**). i. KHMDS, THF,0 °C to rt, TMSCl, 0 °C, **276**, −78°C to rt, then 1 N HCl, 0 °C to rt, 45%; ii. Ph_3_P, imidazole, I_2_, 99%; iii. Ph_3_P; MeCN; (**B**). i. 2-methoxypropene, PPTS, EtOAc, 0 °C to rt, 68%; ii. *n*BuLi, THF, −78 °C, 80%; iii. Dess–Martin oxidation, CH_2_Cl_2_, rt.

**Scheme 38:**
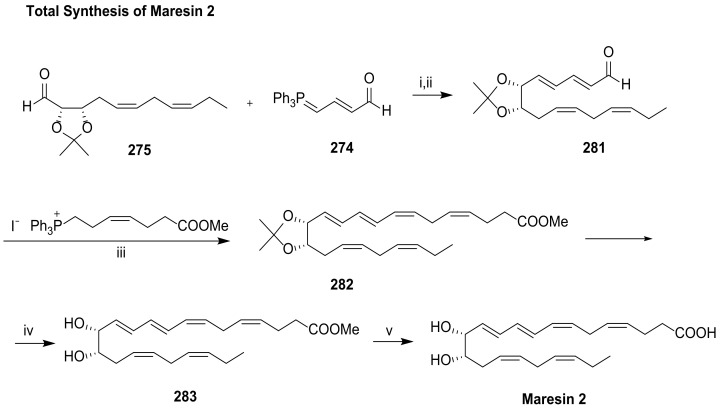
Synthesis of maresin 2, as reported by Rodríguez and Spur [64]. Reagents and conditions: i. CH_2_Cl_2_, rt, 37%; ii. cat. I_2_, benzene, rt, 88%; iii. KHMDS, THF, –78 °C to 0 °C, 37%; iv. PTSA, CH_3_OH, 0 °C, 72%; v. 1 N LiOH, CH_3_OH, H_2_O, 0 °C, then sat. NaH_2_PO_4_, 87%.

The first total synthesis of maresin 2 was carried out by Rodríguez and Spur in 2015 [64]. As shown in Scheme 36, Wittig olefination is the key reaction, assembling molecule fragments for building its core structure. The chirality centres were introduced using a chiral pool strategy starting from 2-deoxy-d-*erythro*-pentopyranose (**256**). The preparation of the key intermediates **273**, **274**, and **275** is illustrated in Scheme 37 and Scheme 38. 

The phosphonium iodide **273** was prepared from aldehyde **277** via *cis*-selective Wittig reaction with hydroxypropylphosphonium bromide, furnishing **278**, the iodination and treatment of which with triphenylphosphane afforded **273** in very high yield (Scheme 37A). The synthesis of C12-C22 precursor **275** (Scheme 37B) was accomplished from **256** through *cis-*diol protection with the group isopropylidene, followed by reaction with the (3*Z*)-3-(hexen-1-ylidene)triphenylphosphorane generated in situ by basic treatment of the phosphonium iodide, providing the (*Z,Z*)-diene **280** in high yield. Dess–Martin oxidation [114] of the primary alcohol afforded the target aldehyde **275**. The Wittig reaction with phosphorane **274** provided aldehyde **281**, which by Wittig *cis*-olefination afforded **282**, the precursor transformed in maresin 2 (Scheme 38) by acid hydrolysis of the propylidene group followed by basic ester hydrolysis.

### 5.3. Maresin-Like Lipid Mediators

**Scheme 39:**
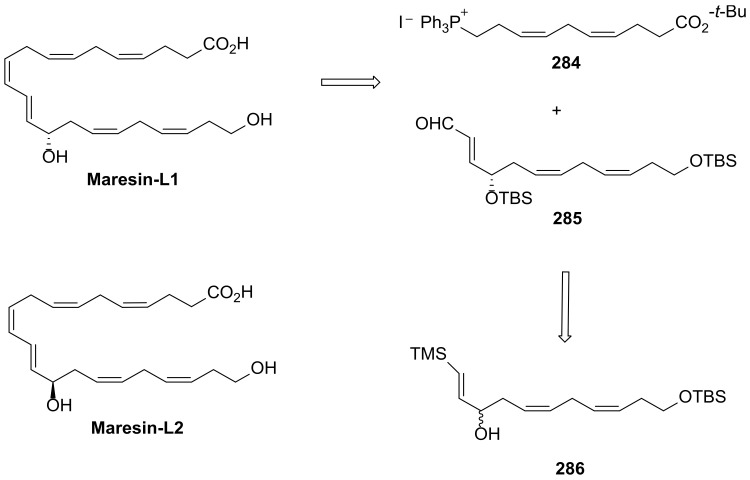
Structure of maresin-L1 and maresin-L2 and retrosynthesis of maresin-L1, as reported by Hong et al. [116].

Discovered in 2014, maresin-L1 (14*S*,22-dihydroxydocosa-4*Z*,7*Z*,10*Z*,12*E*,16*Z*,19*Z*-hexaenoic acid (14*S*,22-diHDHA) and its enantiomer maresin-L2 (14*R*,22-diHDHA) [115] (Scheme 39) are produced by macrophages from DHA [116]. Similar to maresins, their biosynthesis involves 12- or 15-lipoxygenase-catalyzed (14*S*)-hydroxylation, while for maresin-L2 the (14*R*)-hydroxylation is catalysed by cytochrome P450. Maresins-like mediators have a biosynthetic pathway involving a hydroxylation by P450 enzyme(s) at C22 of the polyunsaturated linear chain containing six double bonds and twenty-two carbons. Maresin-Ls act as autocrine/paracrine factors for the reparative functions of leukocytes and PLT in wounds and ameliorate macrophage inflammatory activation, suppressing the chronic inflammation in diabetic wounds caused by activation of macrophages [116]. Thus, these lipid mediators may contribute to the roles of macrophages in wound healing and might be able to be used to restore the impaired reparative function of macrophages for the healing of diabetic wounds [116].

**Scheme 40:**
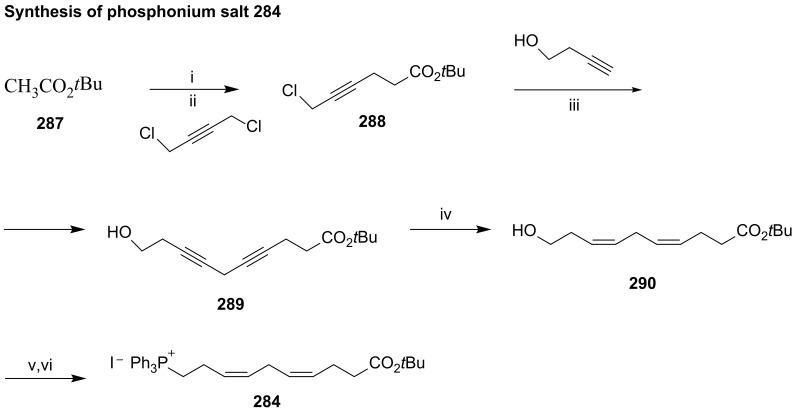
Synthesis of phosphonium salt **284**, as reported by Hong et al. [116]. Reagents and conditions: i. LDA (1.3 equiv.); ii. HMPA (1 equiv.), THF, 0 °C, 1 h; iii. CuI (1.2 equiv.), NaI (1.2 equiv.), Cs_2_CO_3_ (1.2 equiv.), DMF, rt, overnight; iv. H_2_, P-2 Ni (1.2 equiv.), (H_2_NCH_2_)_2_ (2 equiv.), MeOH, 0 °C, 4 h, 80%; v. I_2_ (1.4 equiv.), PPh_3_ (2.5 equiv.), imidazole (2.5 equiv.), CH_2_Cl_2_, rt, 1 h; vi. PPh_3_ (1 equiv.), MeCN, Δ, overnight, 88%.

**Scheme 41:**
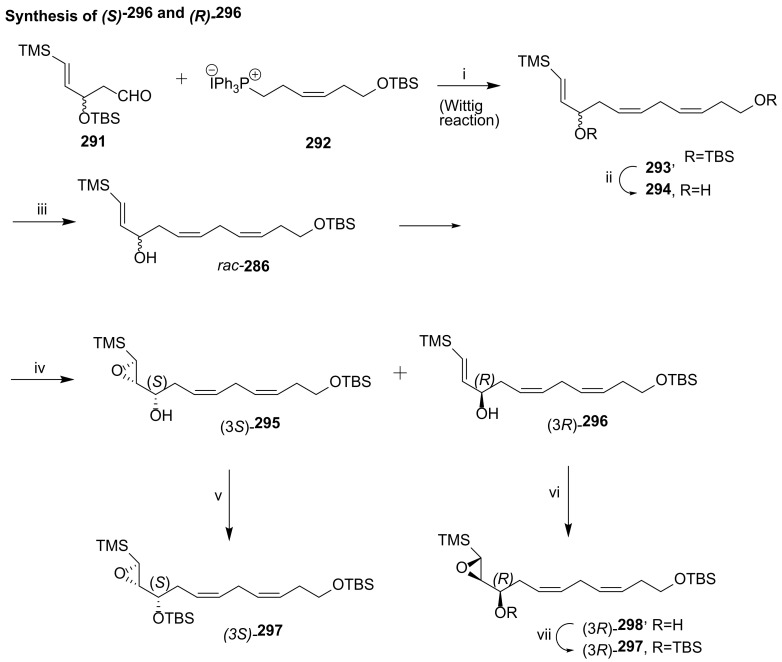
Synthesis of (3*S*)- and (3*R*)-**296**, as reported by Hong et al. [116]. Reagents and conditions: i. **292** (1.1 equiv.), NaN(TMS)_2_ (1.0 equiv.), THF, −78 °C, rt, 15 min; ii. *N*-Bu_4_NF (6 equiv.), THF, rt, 3 h; iii. TBSCl (2 equiv), imidazole (3 equiv), CH_2_Cl_2_, rt, 30min; iv. *t*-BuOOH (1.5 equiv), Ti(O-*i*-Pr)_4_ (1.0 equiv.), l-(+)-DIPT(1.2 equiv.), CH_2_Cl_2_,−18 °C, 5 h; v. TBSOTf (1.5 equiv.), 2,6-lutidine (3 equiv.), CH_2_Cl_2_, 10 min, 97%; vi. *t*BuOOH (1.5 equiv.), Ti(O-*i*-Pr)_4_ (1.0 equiv.), l-(+)-DIPT (1.2 equiv.), CH_2_Cl_2_,−18 °C, 3 h; vii. TBSOTf, 2,6-lutidine, CH_2_Cl_2_, 10 min, 97%.

In 2019, the first stereoselective synthesis of maresin-like lipid mediators (MLs) was disclosed [116]. The key steps are the Wittig reaction of aldehyde **285** with the phosphorane derived from **284** and the kinetic resolution of **286** by asymmetric epoxidation, presented in Scheme 39. The preparation of phosphonium salt **284** was achieved by alkylation of *tert*-butyl acetate to afford **288**, which was coupled with vinyl alcohol to give hydroxydiyneester **290** (Scheme 40). Partial hydrogenation of the triple bond with P-2 nickel afforded hydroxy diene ester **291**, which was iodinated and treated with triphenylphosphane, furnishing phosphonium iodide **284**. The epoxides (3*S*)-**297** and (3*R*)-**297** are the precursors for the synthesis of maresin-L1 and maresin-L2. They were synthesized from racemic aldehyde **291** by a five- and a six-step sequence, respectively (Scheme 41). The first step is the stereoselective Wittig reaction with the phosphorane derived from phosphonium salt **292** by treatment with the base NaN(SiMe_3_)_2_. Desilylation afforded diol **294**, which was then regioselectively silylated at the primary position, furnishing rac-**286**. Sharpless asymmetric epoxidation with l-(+)-DIPT/Ti(O-*i*-Pr)4 afforded epoxy alcohol (3*S*)-**295** with more than 99% ee and (3*R*)-**296** with 91% ee. Silylation of (3*S*)-**295** gave the precursor epoxide (3*S*)-**297** for the synthesis of maresin-L1, while (3*R*)-**296** was converted to epoxide (3*R*)-**298** using the d-(–)-DIPT/Ti(O-*i*Pr)4, followed by silylation to furnish (3*R*)-**297**, the precursor for the preparation of maresin-L2.

**Scheme 42:**
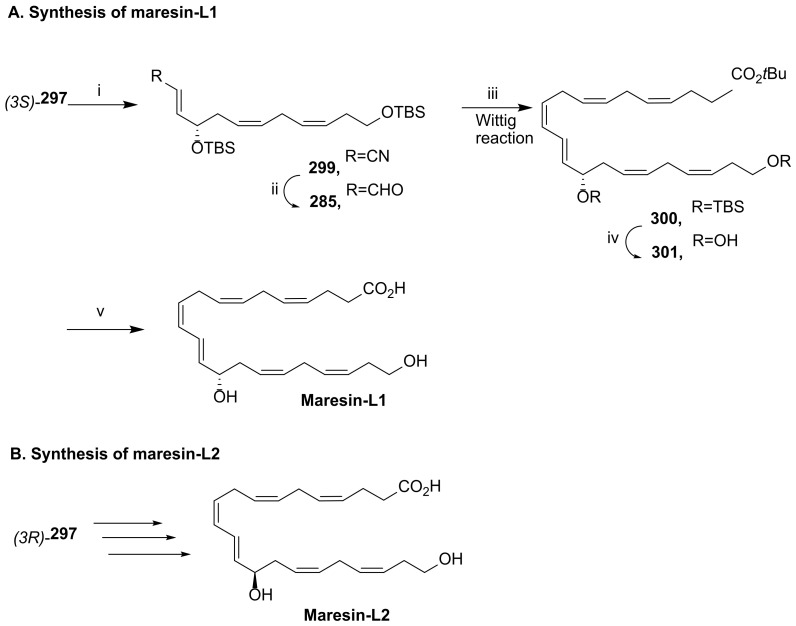
Synthesis of maresin-L1 and maresin-L2, as reported by Hong et al. [116]. Reagents and conditions: i. Et_2_AICN (7 equiv.), THF, rt, 15 min, 95%; ii. DIBAL (2 equiv.), THF, −78°C, rt, 10 min, 75%; iii. **284** (2.8 equiv), NaN (TMS)_2_ (2.7 equiv.), THF, −90 °C to 0 °C,10 min; iv. TBAF (8 equiv.), **301** (84%), THF, rt, 30 min; v. LiOH (30 equiv.), MeOH/H_2_O (1:1), rt, 22 h, 59%.

The synthesis of maresins was accomplished in five steps from the corresponding epoxides. For example, maresin-L1 was synthesized from (3*S*)-**297**, which reacted with diethylaluminum cyanide to afford nitrile 299, which was reduced to aldehyde **285** by DIBAL. Wittig reaction with **284**, desilylation with TBAF, and ester hydrolysis gave maresin-L1 in 35.3% overall yield from (3*S*)-**297**, Scheme 42.

## 6. The New Coronavirus (SARS-CoV-2) and DHA-Derived SPMs

The viral life cycle includes the various stages involved in viral replication that occur inside cells. Thus, it can include three stages: (1) entry, (2) genome replication, and (3) exit. Studies of SPMs as regulators of the inflammatory response caused by viral infections have been carried out, but the effect of SPM on the viral life cycle needs more research.

Coronavirus disease (COVID-19) is caused by the new virus SARS-CoV-2 (severe acute respiratory syndrome coronavirus), which emerged in 2019 in Wuhan (Hubei province in China) and was declared by WHO as a global epidemic. Among risk factors for SARS-CoV-2 infection, obesity contributes with more than 60% of incidence and seems to be related to a deficiency of SPMs [117].

The main symptoms of virus infection are headache, fever, fatigue, cough, and chest tightness, among others [118]. However, this virus can be more aggressive, provoking severe symptoms that include systemic inflammatory response, organ failure, and death [101]. So far, there is no treatment for COVID-19. However, the scientific community has been struggling to offer immune protection against this disease. This effort resulted in more than 300 vaccine projects, with some of them already approved, such as a Pfizer–BioNTech vaccine, produced in US, and an AstraZeneca vaccine, developed by the University of Oxford and produced in UK, Sweden, and Italy [119], among others.

COVID-19 severe cases are much more common in elderly people with other morbidities, such as diabetes, cardiovascular diseases, hypertension, and respiratory diseases [117]. SARS-CoV-2 is an RNA virus that infects the lungs and consequently cells of the immune system that try to react to the inflammation. Among the human body immune system responses to COVID-19, excessive stimulation of the inflammatory response can occur, which is called “cytokine storm”, indicating a state of hyperinflammation and resulting in dysregulation of lipid transport. The DHA metabolites resolvins, protectins, and maresins behave as “turn off switch” in inflammatory processes and play an important role in viral infections [117]. The resolvins of D series, particularly D1, D2, D3, D4, and D5 and the intermediate 17HDHA may have a functional role as lipid mediators in the “cytokine storm” of SARS-CoV-2 infection, diminishing pro-inflammatory levels [120,121]. Moreover, RvD2 and MaR1 are described as block inflammasome components, leading to the reduction in IL-1β release, a potent pro-inflammatory cytokine [39].

It is known that DHA inactivates enveloped viruses and can inhibit proliferation of some microbial organisms [120,122]; as described by Torrinhas et al. [121], DHA supplementation can improve blood oxygenation in patients with acute respiratory distress syndrome, with a consequent reduction in ventilation requirement and length of stay in the ICU [105]. Additionally, patients receiving parenteral nutrition therapy fortified with lipid emulsion enriched in DHA were reported to have decreased infection and sepsis risk and a reduction in ICU stay of about 2 days [121]. Despite requiring further research, the consumption of DHA in the diet seems to help improve treatment and recovery of severe COVID-19, as DHA-SPMs are antiinflammation pro-resolving agents [123,124].

Another potential site for the entrance of SARS-CoV-2 in the human body is through the cornea, where the SARS-CoV-2 receptors are expressed, including ACE2 and host proteases for the S protein. Thus, recent studies [61] pointed out that DHA-SPMs such as RvD6 isomers and elovanoid N32 reduced the expression of angiotensin converting enzyme 2 (ACE2). On the other hand, NDP1 did not reduce it. RvD6 isomers and elovanoid N32 also counteract the binding of the receptor-binding domain of SARS-CoV-2 spike (S) protein to the injured cornea [125].

Further research is needed on the role of RvDs, PDs, and MaRs in SARsCOV-2 and the effect of DHA-SPM on the viral life cycle. Moreover, the interaction between the host immune system and infectious viral attack is also a very promising research area. Thus, the bioactivity of these lipid mediators will contribute to open therapeutic avenues to counteract virus attachment and entrance to the human body.

## 7. Conclusions

This review summarizes current knowledge on DHA metabolites as anti-inflammatory lipid mediators with pro-resolving activity. DHA can be obtained essentially from marine sources and is available at sites of acute inflammation, where it is converted in diverse bioactive metabolites—namely, the SPM D-series resolvins, protectins, and maresins that act at subnanomolar doses. SPMs are produced in human body fluids and organs and are involved in the resolution of inflammation, wound healing, and neuroprotection, acting in neutrophils, macrophages, endothelial and epithelial cells, and lymphocytes. DHA-SPMs have several health benefits; in particular, D-resolvins were reported as neuroprotective agents, also acting against cell injury-induced oxidative stress as well as protectins. Maresins show action in nervous system diseases, and they have a powerful protective effect in inflammation, oxidative stress, immune diseases, and tissue regeneration. More recently, with the rise of COVID-19, DHA-SPM_S_—mainly protectins and D-series resolvins—demonstrated relevant action, decreasing cytokine storm and consequently pro-inflammatory levels. In addition, the recently discovered interaction of RvD6 with the binding domain of SARS-CoV-2 spike protein is very promising and encourages further investigation in this area. Although DHA-SPMs structure and biosynthesis is already known, the access to these molecules is scarce from natural resources and by biosynthetic pathways. Their potent bioactivities encourage further investigation aiming at their valorisation for therapeutic purposes, but this research requires access to large amounts of the studied product. These issues motivated the scientific community to develop their total organic synthesis, illustrated in this review for DHA-SPMs. These anti-inflammatory lipid mediators are very complex molecules with defined stereochemistry. Indeed, the highly stereoselective and convergent approaches developed demonstrate the importance of organic chemistry in accessing these molecules towards development of new therapeutics against neurodegenerative diseases, coronary heart disease, cancer, diabetes, and viral infections, opening new avenues for COVID-19 therapy research.

## Figures and Tables

**Table 1:** Nutraceutical potential of omega-3 fatty acids.

Health Benefits of Dietary Supplementation	Reference
Reduce risks of macular degeneration and cancers	[7]
Protect against atherosclerotic heart disease and sudden coronary death	[8]
Have beneficial effect in patients with Meibomian gland dysfunction (MGD)	[9]
Reduce risks of developing cardiovascular disease (CVD), coronary heart disease (CHD), and myocardial infarction (MI)	[10]
Reduce risks of Parkinson’s and Alzheimer’s disease	[11]
Decrease systolic and diastolic blood pressure	[12]
Inhibit pro-inflammatory signalling cascades	[13]

**Table 2:** DHA-derived SPM actions in disease models and cell targets.

SPM	Target	Bioaction	Reference
RvD1	Polymorphonuclear leukocyte	Decreases infiltration in murine skin air pouch model; limits infiltration in renal ischaemic injury	[36,42,43]
	Microglial cells	Inhibits IL-1β expression in vitro	[36]
	Vascular inflammation (arterial angioplasty)	Attenuates cell proliferation, leukocyte recruitment, and neointimal hyperplasia	[44]
	Alzheimer’s disease	Stimulates phagocytosis of Aβ by Alzheimer’s disease macrophages	[45]
	Pain	Controls inflammatory pain	[46]
AT-RvD1	Pain	Controls inflammatory pain	[47]
	Temporomandibular joint inflammation	Limits PMN infiltration to CFA-inflamed TMJ	[48]
	Arthritis	Antihyperalgesic	[49]
	Fibromyalgia	Reduces mechanical allodynia and thermal sensitization and prevents depressive behaviour	[50]
	Postsurgical cognitive decline	Improves postoperative decline and attenuates memory	[51]
PD1	Polymorphonuclear leukocyte	Upregulates CCR5 expression; reduces tissue infiltration	[52,53]
	Macrophages	Stimulates phagocytosis of apoptotic polymorphonuclear leukocyte	[54]
	T Cell	Promotes apoptosis in vitro	[55]
	Glial cells	Reduces cytokine production	[56]
	Epithelial cells	Protects from oxidative stress-induced apoptosis (retinal pigment epithelium)	[38]
	Eosinophils	Decreases recruitment in response to allergen	[57]
RvD2	Macroglia	Prohibits or reduces the activation of macroglia and microglia, respectively	[58]
	-	Downregulates TNF-α, IL-1β, iNOS, NF-k, NO, and ROS production	[35]
	Burn wound	Prevents secondary thrombosis and necrosis	[48]
RvD3 and AT-RvD3	Neutrophils	Regulates neutrophils and mediators, reducing murine peritonitis and dermal inflammation	[59]
RvD4	Protection and resolution of inflammation during bacterial infection	Stops leukocyte influx to the site of infection in the dorsal pouch cavity as well as the inflammation-initiating eicosanoids by reducing levels of PGD_2_ and LTB_4_	[60]
RvD5	Bacterial infection	Increases survival and lower antibiotic requirement	[48]
RvD6	Corneal nerve	Decreases inflammation and increases wound healing and nerve regeneration by decreasing the expression of the ACE2 receptor, furin, and integrins.	[61]
MaR1	Pain	Controls inflammatory pain	[37]
	Tissue regeneration	Promotes tissue regeneration in planaria	[37]
	Neuroprotection	Treats amyotrophic lateral sclerosis and spinal muscular atrophy Accelerates clearance of neutrophils and a reduction in macrophage accumulation at the lesion site	[62,63]
MaR2	Macrophage phagocytosis	Anti-inflammatory activity	[64]

## Data Availability

Not applicable.

## References

[1] Aneiros A., Garateix A. (2004). Bioactive peptides from marine sources: Pharmacological properties and isolation procedures. J. Chromatogr. B Anal. Technol. Biomed. Life Sci..

[2] Petersen L.-E., Kellermann M.Y., Schupp P.J. (2020). Secondary Metabolites of Marine Microbes: From Natural Products Chemistry to Chemical Ecology. YOUMARES.

[3] Blunt J.W., Copp B.R., Keyzers R.A., Munro M.H.G., Prinsep M.R. (2017). Marine natural products. Nat. Prod. Rep..

[4] Gallimore W. (2017). Marine Metabolites: Oceans of Opportunity. Pharmacogn. Fundam. Appl. Strategy.

[5] Barzkar N., Jahromi S.T., Poorsaheli H.B., Vianello F. (2019). Metabolites from marine microorganisms, micro, and macroalgae: Immense scope for pharmacology. Mar. Drugs.

[6] Hamed I., Özogul F., Özogul Y., Regenstein J.M. (2015). Marine Bioactive Compounds and Their Health Benefits: A Review. Compr. Rev. Food Sci. Food Saf..

[7] Zhang G., Panigrahy D., Mahakian L.M., Yang J., Liu J.Y., Lee K.S., Wettersten H.I., Ulu A., Hu X., Tam S. (2013). Epoxy metabolites of docosahexaenoic acid (DHA) inhibit angiogenesis, tumor growth, and metastasis. Proc. Natl. Acad. Sci. USA.

[8] Mori T.A., Beilin L.J. (2004). Omega-3 fatty acids and inflammation. Curr. Atheroscler. Rep..

[9] Oleñik A., Mahillo-Fernández I., Alejandre-Alba N., Fernández-Sanz G., Alarcón Pérez M., Luxan S., Quintana S., Martínez de Carneros Llorente A., García-Sandoval B., Jiménez-Alfaro I. (2014). Benefits of omega-3 fatty acid dietary supplementation on health-related quality of life in patients with meibomian gland dysfunction. Clin. Ophthalmol..

[10] Innes J.K., Calder P.C. (2020). Marine omega-3 (N-3) fatty acids for cardiovascular health: An update for 2020. Int. J. Mol. Sci..

[11] Avallone R., Vitale G., Bertolotti M. (2019). Omega-3 fatty acids and neurodegenerative diseases: New evidence in clinical trials. Int. J. Mol. Sci..

[12] Tur J.A., Bibiloni M.M., Sureda A., Pons A. (2012). Dietary sources of omega 3 fatty acids: Public health risks and benefits. Br. J. Nutr..

[13] Calder P.C. (2010). Omega-3 fatty acids and inflammatory processes. Nutrients.

[14] Cardoso C., Afonso C., Bandarra N.M. (2018). Dietary DHA, bioaccessibility, and neurobehavioral development in children. Crit. Rev. Food Sci. Nutr..

[15] Bandarra N.M., Batista I., Nunes M.L., Empis J.M., Christie W.W. (1997). Seasonal changes in lipid composition of sardine (*Sardina pilchardus*). J. Food Sci..

[16] Ferreira I., Gomes-Bispo A., Lourenço H., Matos J., Afonso C., Cardoso C., Castanheira I., Motta C., Prates J.A.M., Bandarra N.M. (2020). The chemical composition and lipid profile of the chub mackerel (Scomber colias) show a strong seasonal dependence: Contribution to a nutritional evaluation. Biochimie.

[17] Welch A.A., Shakya-Shrestha S., Lentjes MA H., Wareham N.J., Khaw K.T. (2010). Dietary intake and status of n-3 polyunsaturated fatty acids in a population of fish-eating and non-fish-eating meat-eaters, vegetarians, and vegans and the precursor-product ratio of α-linolenic acid to long-chain n-3 polyunsaturated fatty acids: Results. Am. J. Clin. Nutr..

[18] Cardoso C., Afonso C., Bandarra N.M. (2016). Dietary DHA and health: Cognitive function ageing. Nutr. Res. Rev..

[19] Calder P.C. (2016). Docosahexaenoic acid. Ann. Nutr. Metab..

[20] Kuratko C.N., Barrett E.C., Nelson E.B., Salem N. (2013). The relationship of docosahexaenoic acid (DHA) with learning and behavior in healthy children: A review. Nutrients.

[21] Martins B.P., Bandarra N.M., Figueiredo-Braga M. (2020). The role of marine omega-3 in human neurodevelopment, including Autism Spectrum Disorders and Attention-Deficit/Hyperactivity Disorder–a review. Crit. Rev. Food Sci. Nutr..

[22] Nguyen Q.V., Malau-Aduli B.S., John C., Nichols P.D., Malau-Aduli E.O. (2019). Enhancing Omega-3 Long-Chain Polyunsaturated Human Consumption. Nutrients.

[23] Kwon Y. (2020). Immuno-Resolving Ability of Resolvins, Protectins, and Maresins Derived from Omega-3 Fatty Acids in Metabolic Syndrome. Mol. Nutr. Food Res..

[24] Kuda O. (2017). Bioactive metabolites of docosahexaenoic acid. Biochimie.

[25] Jaén R.I., Sánchez-García S., Fernández-Velasco M., Boscá L., Prieto P. (2021). Resolution-Based Therapies: The Potential of Lipoxins to Treat Human Diseases. Front. Immunol..

[26] Duvall M.G., Levy B.D. (2016). DHA- and EPA-derived resolvins, protectins, and maresins in airway inflammation. Eur. J. Pharmacol..

[27] Serhan C.N., Levy B.D. (2018). Resolvins in inflammation: Emergence of the pro-resolving superfamily of mediators. J. Clin. Investig..

[28] Tungen J.E., Aursnes M., Hansen T.V. (2015). Stereoselective synthesis of maresin 1. Tetrahedron Lett..

[29] Serhan C.N., Petasis N.A. (2011). Resolvins and protectins in inflammation resolution. Chem. Rev..

[30] Malawista S.E., Chevance AD B., Van Damme J., Serhan C.N. (2008). Tonic inhibition of chemotaxis in human plasma. Proc. Natl. Acad. Sci. USA.

[31] Gordon S. (2016). Phagocytosis: An Immunobiologic Process. Immunity.

[32] Serhan C.N., Chiang N. (2013). Resolution phase lipid mediators of inflammation: Agonists of resolution. Curr. Opin. Pharmacol..

[33] Serhan C.N. (2017). Treating inflammation and infection in the 21st century: New hints from decoding resolution mediators and mechanisms. FASEB J..

[34] Serhan C.N. (2014). Pro-resolving lipid mediators are leads for resolution physiology. Nature.

[35] Li C., Wu X., Liu S., Shen D., Zhu J., Liu K. (2020). Role of Resolvins in the Inflammatory Resolution of Neurological Diseases. Front. Pharmacol..

[36] Serhan C.N., Hong S., Gronert K., Colgan S.P., Devchand P.R., Mirick G., Moussignac R.-L. (2002). Resolvins: A family of bioactive products of omega-3 fatty acid transformation circuits initiated by aspirin treatment that counter proinflammation signals. J. Exp. Med..

[37] Serhan C.N., Dalli J., Karamnov S., Choi A., Park C., Xu Z., Ji R., Zhu M., Petasis N.A. (2012). Macrophage proresolving mediator maresin 1 stimulates tissue regeneration and controls pain. FASEB J..

[38] Mukherjee P.K., Marcheselli V.L., Serhan C.N., Bazan N.G. (2004). Neuroprotectin D1: A docosahexaenoic acid-derived docosatriene protects human retinal pigment epithelial cells from oxidative stress. Proc. Natl. Acad. Sci. USA.

[39] Chiang N., Serha C.N. (2020). Specialized pro-resolving mediator network: An update on production and actions. Essays Biochem..

[40] Han Y.H., Lee K., Saha A., Han J., Choi H., Noh M., Lee Y.H., Lee M.O. (2021). Specialized proresolving mediators for therapeutic interventions targeting metabolic and inflammatory disorders. Biomol. Ther..

[41] Ariyoshi T., Hagihara M., Eguchi S., Fukuda A., Iwasaki K., Oka K., Takahashi M., Yamagishi Y., Mikamo H. (2020). Clostridium butyricum MIYAIRI 588-Induced Protectin D1 Has an Anti-inflammatory Effect on Antibiotic-Induced Intestinal Disorder. Front. Microbiol..

[42] Duffield J.S., Hong S., Vaidya V.S., Lu Y., Fredman G., Serhan C.N., Bonventre J.V. (2006). Resolvin D Series and Protectin D1 Mitigate Acute Kidney Injury. J. Immunol..

[43] Kohli P., Levy B.D. (2009). Resolvins and protectins: Mediating solutions to inflammation. Br. J. Pharmacol..

[44] Miyahara T., Runge S., Chatterjee A., Chen M., Mottola G., Fitzgerald J.M., Serhan C.N., Conte M.S. (2013). D-series resolvin attenuates vascular smooth muscle cell activation and neointimal hyperplasia following vascular injury. FASEB J..

[45] Mizwicki M.T., Liu G., Fiala M., Magpantay L., Sayre J., Siani A., Mahanian M., Weitzman R., Hayden E.Y., Rosenthal M.J. (2013). 1α,25-dihydroxyvitamin D3 and resolvin D1 retune the balance between amyloid-β phagocytosis and inflammation in Alzheimer’s disease patients. J. Alzheimer’s Dis..

[46] Xu Z.Z., Zhang L., Liu T., Park J.Y., Berta T., Yang R., Serhan C.N., Ji R.R. (2010). Resolvins RvE1 and RvD1 attenuate inflammatory pain via central and peripheral actions. Nat. Med..

[47] Bang S., Yoo S., Yang T.J., Cho H., Hwang S. (2012). 17(R)-resolvin D1 specifically inhibits transient receptor potential ion channel vanilloid 3 leading to peripheral antinociception. Br. J. Pharmacol..

[48] Serhan C.N., Chiang N., Dalli J., Levy B.D., Tracey K.J., Serhan C.N., Chiang N., Dalli J., De Zoete M.R., Palm N.W. (2014). Lipid mediators in the resolution of inflammation. Cold Spring Harb. Perspect. Biol..

[49] Lima-Garcia J.F., Dutra R.C., Silva D.A., Motta E.M., Campos M.M., Calixto J.B. (2011). The precursor of resolvin D series and aspirin-triggered resolvin D1 display anti-hyperalgesic properties in adjuvant-induced arthritis in rats. Br. J. Pharmacol..

[50] Klein C.P., Sperotto ND M., Maciel I.S., Leite C.E., Souza A.H., Campos M.M. (2014). Effects of D-series resolvins on behavioral and neurochemical changes in a fibromyalgia-like model in mice. Neuropharmacology.

[51] Terrando N., Gómez-Galán M., Yang T., Carlström M., Gustavsson D., Harding R.E., Lindskog M., Eriksson L.I. (2013). Aspirin-triggered resolvin D1 prevents surgery-induced cognitive decline. FASEB J..

[52] Marcheselli V.L., Hong S., Lukiw W.J., Tian X.H., Gronert K., Musto A., Hardy M., Gimenez J.M., Chiang N., Serhan C.N. (2003). Novel Docosanoids Inhibit Brain Ischemia-Reperfusion-mediated Leukocyte Infiltration and Pro-inflammatory Gene Expression. J. Biol. Chem..

[53] Ariel A., Fredman G., Sun Y.P., Kantarci A., Dyke TE V.a.n., Luster A.D., Serhan C.N. (2006). Apoptotic neutrophils and T cells sequester chemokines during immune response resolution through modulation of CCR5 expression. Nat. Immunol..

[54] Schwab J.M., Chiang N., Arita M., Serhan C.N. (2007). Resolvin E1 and protectin D1 activate inflammation-resolution programmes. Nature.

[55] Ariel A., Li P.L., Wang W., Tang W.X., Fredman G., Hong S., Gotlinger K.H., Serhan C.N. (2005). The docosatriene protectin D1 is produced by TH2 skewing promotes human T cell via lipid raft clustering. J. Biol. Chem..

[56] Hong S., Gronert K., Devchand P.R., Moussignac R.L., Serhan C.N. (2003). Novel docosatrienes and 17S-resolvins generated from docosahexaenoic acid in murine brain, human blood, and glial cells: Autacoids in anti-inflammation. J. Biol. Chem..

[57] Levy B.D., Kohli P., Gotlinger K., Haworth O., Hong S., Kazani S., Israel E., Haley K.J., Serhan C.N. (2007). Protectin D1 Is Generated in Asthma and Dampens Airway Inflammation and Hyperresponsiveness. J. Immunol..

[58] Tian Y., Zhang Y., Zhang R., Qiao S., Fan J. (2015). Resolvin D2 recovers neural injury by suppressing inflammatory mediators expression in lipopolysaccharide-induced Parkinson’s disease rat model. Biochem. Biophys. Res. Commun..

[59] Dalli J., Winkler J.W., Colas R.A., Arnardottir H., Cheng CY C., Chiang N., Petasis N.A., Serhan C.N. (2013). Resolvin D3 and aspirin-triggered resolvin D3 are potent immunoresolvents. Chem. Biol..

[60] Winkler J.W., Orr S.K., Dalli J., Cheng C.Y., Sanger J.M., Chiang N., Petasis N.A., Serhan C.N. (2016). Resolvin D4 stereoassignment and its novel actions in host protection and bacterial clearance. Sci. Rep..

[61] Pham T.L., Kakazu A.H., He J., Nshimiyimana R., Petasis N.A., Jun B., Bazan N.G., Bazan HE P. (2021). Elucidating the structure and functions of Resolvin D6 isomers on nerve regeneration with a distinctive trigeminal transcriptome. FASEB J..

[62] Hwang S.M., Chung G., Kim Y.H., Park C.K. (2019). The role of maresins in inflammatory pain: Function of macrophages in wound regeneration. Int. J. Mol. Sci..

[63] Francos-Quijorna I., Santos-Nogueira E., Gronert K., Sullivan A.B., Kopp M.A., Brommer B., David S., Schwab J.M., López-Vales R. (2017). Maresin 1 promotes inflammatory resolution, neuroprotection, and functional neurological recovery after spinal cord injury. J. Neurosci..

[64] Rodriguez A.R., Spur B.W. (2015). First total synthesis of the macrophage derived anti-inflammatory and pro-resolving lipid mediator Maresin 2. Tetrahedron Lett..

[65] Dangi B., Obeng M., Nauroth J.M., Teymourlouei M., Needham M., Raman K., Arterburn L.M. (2009). Biogenic synthesis, purification, and chemical characterization of anti-inflammatory resolvins derived from docosapentaenoic acid (DPAn-6). J. Biol. Chem..

[66] Oh S.F., Vickery T., Schmidt B.A., Serhan C.N., Chiang N., Fredman G., Ba F. (2012). Infection regulates pro-resolving mediators that lower antibiotic requirements. Nature.

[67] Oh S.F., Pillai P.S., Recchiuti A., Yang R., Serhan C.N. (2011). Pro-resolving actions and stereoselective biosynthesis of 18S E-series resolvins in human leukocytes and murine inflammation. J. Clin. Investig..

[68] Hong S., Lu Y., Yang R., Gotlinger K.H., Petasis N.A., Serhan C.N. (2007). Resolvin D1, Protectin D1, and related docosahexaenoic acid-derived products: Analysis via electrospray/low energy tandem mass spectrometry based on spectra and fragmentation mechanisms. J. Am. Soc. Mass Spectrom..

[69] Bazan N.G. (2005). Neuroprotectin D1 (NPD1): A DHA-derived mediator that protects brain and retina against cell injury-induced oxidative stress. Brain Pathol..

[70] Chiang N., Serhan C.N. (2017). Structural elucidation and physiologic functions of specialized pro-resolving mediators and their receptors. Mol. Asp. Med..

[71] Brennan E., Kantharidis P., Cooper M.E. (2021). Pro-resolving lipid mediators: Regulators of inflammation, metabolism and kidney function. Nat. Rev. Nephrol..

[72] Chen J., Shetty S., Zhang P., Gao R., Hu Y., Wang S., Li Z., Fu J. (2014). Aspirin-triggered resolvin D1 down-regulates inflammatory responses and protects against endotoxin-induced acute kidney injury. Toxicol. Appl. Pharmacol..

[73] Luan H., Wang C., Sun J., Zhao L., Li L., Zhou B., Shao S., Shen X., Xu Y. (2020). Resolvin D1 Protects Against Ischemia/Reperfusion-Induced Acute Kidney Injury by Increasing Treg Percentages via the ALX/FPR2 Pathway. Front. Physiol..

[74] Qu X., Zhang X., Yao J., Song J., Nikolic-Paterson D.J., Li J. (2012). Resolvins E1 and D1 inhibit interstitial fibrosis in the obstructed kidney via inhibition of local fibroblast proliferation. J. Pathol..

[75] Sulciner M.L., Serhan C.N., Gilligan M.M., Mudge D.K., Chang J., Gartung A., Lehner K.A., Bielenberg D.R., Schmidt B., Dalli J. (2018). Resolvins suppress tumor growth and enhance cancer therapy. J. Exp. Med..

[76] Videla L.A., Vargas R., Valenzuela R., Muñoz P., Corbari A., Hernandez-Rodas M.C. (2019). Combined administration of docosahexaenoic acid and thyroid hormone synergistically enhances rat liver levels of resolvins RvD1 and RvD2. Prostaglandins Leukot. Essent. Fat. Acids.

[77] Hansen T.V., Vik A., Serhan C.N. (2019). The protectin family of specialized pro-resolving mediators: Potent immunoresolvents enabling innovative approaches to target obesity and diabetes. Front. Pharmacol..

[78] Gilligan M.M., Gartung A., Sulciner M.L., Norris P.C., Sukhatme V.P., Bielenberg D.R., Huang S., Kieran M.W., Serhan C.N., Panigrahy D. (2019). Aspirin-triggered proresolving mediators stimulate resolution in cancer. Proc. Natl. Acad. Sci. USA.

[79] Sun Y.P., Oh S.F., Uddin J., Yang R., Gotlinger K., Campbell E., Colgan S.P., Petasis N.A., Serhan C.N. (2007). Resolvin D1 and its aspirin-triggered 17R epimer: Stereochemical assignments, anti-inflammatory properties, and enzymatic inactivation. J. Biol. Chem..

[80] Rodriguez A.R., Spur B.W. (2012). Total synthesis of Resolvin D1, a potent anti-inflammatory lipid mediator. Tetrahedron Lett..

[81] Morita M., Wu S., Kobayashi Y. (2019). Stereocontrolled synthesis of resolvin D1. Org. Biomol. Chem..

[82] Colvin W., Hamill J. (1973). One- step Conversion of Carbonyl Compounds into Acetylenes By ERNEST. Chem. Commun..

[83] Kazuhiro Miwa T.A.T.S. (1993). Extension of the colvin rearrangement using trimethylsilyldiazomethane. A new synthesis of alkynes. Synlett.

[84] Huang Z., Negishi E.I. (2006). A convenient and genuine equivalent to HZrCp2Cl generated in situ from ZrCp2Cl2-DIBAL-H. Org. Lett..

[85] Rodríguez A.R., Spur B.W. (2004). First total synthesis of 7(S),16(R),17(S)-Resolvin D2, a potent anti-inflammatory lipid mediator. Tetrahedron Lett..

[86] Li J., Leong M.M., Stewart A., Rizzacasa M.A. (2013). Total synthesis of the endogenous inflammation resolving lipid resolvin D2 using a common lynchpin. Beilstein J. Org. Chem..

[87] Rodríguez A., Nomen M., Spur B.W., Godfroid J.J. (1999). Selective oxidation of primary silyl ethers and its application to the synthesis of natural products. Tetrahedron Lett..

[88] Urbitsch F., Elbert B.L., Llaveria J., Streatfeild P.E., Anderson E.A. (2020). A Modular, Enantioselective Synthesis of Resolvins D3, E1, and Hybrids. Org. Lett..

[89] Soullez D., Plé G., Duhamel L. (1997). ω-Halogeno polyenals: Preparation and application to a one-pot synthesis of polyenals from carbonyl compounds. J. Chem. Soc. Perkin Trans..

[90] Winkler J.W., Libreros S., La Rosa X.D.e., Sansbury B.E., Norris P.C., Chiang N., Fichtner D., Keyes G.S., Wourms N., Spite M. (2018). Frontline Science: Structural insights into Resolvin D4 actions and further metabolites via a new total organic synthesis and validation. J. Leukoc. Biol..

[91] Spur B.W., Rodrı A.R. (2005). First total synthesis of 7(S), 17(S)-Resolvin D5, a potent anti-inflammatory docosanoid. Tetrahedron Lett..

[92] Takai K., Nitta K., Utimoto K. (1986). Simple and selective method for aldehydes (RCHO). fwdarw. (E)-haloalkenes (RCH:CHX) conversion by means of a haloform-chromous chloride system. J. Am. Chem. Soc..

[93] Ogawa N., Sugiyama T., Morita M., Suganuma Y., Kobayashi Y. (2017). Total Synthesis of Resolvin D5. J. Org. Chem..

[94] Rodriguez A.R., Spur B.W. (2012). First total synthesis of the anti-inflammatory lipid mediator Resolvin D6. Tetrahedron Lett..

[95] Morita M., Tanabe S., Arai T., Kobayashi Y. (2019). Synthesis of Resolvin D6 and the Silyl Ether of the Resolvin E2 Methyl Ester via trans -Enynyl Alcohols. Synlett.

[96] Labarre-Lainé J., Beniazza R., Desvergnes V., Landais Y. (2013). Convergent access to bis-spiroacetals through a sila-Stetter-ketalization cascade. Org. Lett..

[97] Matsumura K., Hashiguchi S., Ikariya T., Noyori R. (1997). The first asymmetric transfer hydrogenation of acetylenic ketones using chiral Ru (II) catalysts and 2-propanol as the hydrogen donor. 9 This method allows highly selective reduction of structurally diverse acetylenic ketones to propargylic alcohols of. J. Am. Chem. Soc..

[98] Aursnes M., Tungen J.E., Vik A., Colas R., Cheng C.C., Dalli J., Serhan C.N., Hansen T. (2015). V Total Synthesis of the Lipid Mediator PD1. J. Nat. Prod..

[99] Asatryan A., Bazan N.G. (2017). Molecular mechanisms of signaling via the docosanoid neuroprotectin D1 for cellular homeostasis and neuroprotection. J. Biol. Chem..

[100] Marcheselli V.L., Mukherjee P.K., Arita M., Hong S., Antony R., Sheets K., Winkler J.W., Petasis N.A., Serhan C.N., Bazan N.G. (2010). Neuroprotectin D1/protectin D1 stereoselective and specific binding with human retinal pigment epithelial cells and neutrophils. Prostaglandins Leukot. Essent. Fat. Acids.

[101] Bazan N.G., Calandria J.M., Serhan C.N. (2010). Rescue and repair during photoreceptor cell renewal mediated by docosahexaenoic acid-derived neuroprotectin D1. J. Lipid Res..

[102] Singh N.K., Rao G.N. (2019). Emerging role of 12/15-Lipoxygenase (ALOX15) in human pathologies. Prog. Lipid Res..

[103] González-Périz A., Horrillo R., Ferré N., Gronert K., Dong B., Morán-Salvador E., Titos E., Martínez-Clemente M., López-Parra M., Arroyo V. (2009). Obesity-induced insulin resistance and hepatic steatosis are alleviated by ω-3 fatty acids: A role for resolvins and protectins. FASEB J..

[104] Clària J., López-Vicario C., Rius B., Titos E. (2017). Pro-resolving actions of SPM in adipose tissue biology. Mol. Asp. Med..

[105] Ogawa N., Kobayashi Y. (2011). Total synthesis of the antiinflammatory and proresolving protectin D1. Tetrahedron Lett..

[106] Petasis N.A., Yang R., Winkler J.W., Zhu M., Uddin J., Bazan N.G., Serhan C.N. (2012). Stereocontrolled total synthesis of Neuroprotectin D1/Protectin D1 and its aspirin-triggered stereoisomer. Tetrahedron Lett..

[107] Rodriguez A.R., Spur B.W. (2014). Total synthesis of the potent anti-inflammatory lipid mediator Protectin D1. Tetrahedron Lett..

[108] Balas L., Risé P., Gandrath D., Rovati G., Bolego C., Stellari F., Trenti A., Buccellati C., Durand T., Sala A. (2019). Rapid Metabolization of Protectin D1 by β-Oxidation of Its Polar Head Chain. J. Med. Chem..

[109] Müller S., Liepold B., Gerald J., Roth H.J.B. (1996). An Improved One-pot Procedure for the Synthesis of Alkynes from Aldehydes. Synlett.

[110] Dayaker G., Durand T., Balas L. (2014). A versatile and stereocontrolled total synthesis of dihydroxylated docosatrienes containing a conjugated E,E,Z-triene. Chem. Eur. J..

[111] Li Q.F., Hao H., Tu W.S., Guo N., Zhou X.Y. (2020). Maresins: Anti-inflammatory pro-resolving mediators with therapeutic potential. Eur. Rev. Med. Pharmacol. Sci..

[112] Sasaki K., Urabe D., Arai H., Arita M., Inoue M. (2011). Total synthesis and bioactivities of two proposed structures of maresin. Chem. Asian J..

[113] Rodriguez A.R., Spur B.W. (2012). Total synthesis of the macrophage derived anti-inflammatory lipid mediator Maresin 1. Tetrahedron Lett..

[114] Dess D.B., Martin J.C. (1991). A Useful 12-I-5 Triacetoxyperiodinane (the Dess-Martin Periodinane) for the Selective Oxidation of Primary or Secondary Alcohols and a Variety of Related 12-I-5 Species. J. Am. Chem. Soc..

[115] Hong S., Lu Y., Tian H., Alapure B.V., Wang Q., Bunnell B.A., Laborde J.M. (2014). Maresin-like lipid mediators are produced by leukocytes and platelets and rescue reparative function of diabetes-impaired macrophages. Chem. Biol..

[116] Hong S., Lu Y., Morita M., Saito S., Kobayashi Y., Jun B., Bazan N.G., Xu X., Wang Y. (2019). Stereoselective Synthesis of Maresin-Like Lipid Mediators. Synlett.

[117] Calder P.C. (2020). Nutrition, immunity and COVID-19. BMJ Nutr. Prev. Health.

[118] Alimohamadi Y., Sepandi M., Taghdir M., Hosamirudsari H. (2020). Determine the most common clinical symptoms in COVID-19 patients: A systematic review and meta-analysis. J. Prev. Med. Hyg..

[119] Forni G., Mantovani A., Forni G., Mantovani A., Moretta L., Rappuoli R., Rezza G., Bagnasco A., Barsacchi G., Bussolati G. (2021). COVID-19 vaccines: Where we stand and challenges ahead. Cell Death Differ..

[120] Simopoulos A.P., Serhan C.N., Bazinet R.P. (2021). The need for precision nutrition, genetic variation and resolution in Covid-19 patients. Mol. Asp. Med..

[121] Torrinhas R.S., Calder P.C., Lemos G.O., Waitzberg D.L. (2021). Parenteral fish oil: An adjuvant pharmacotherapy for coronavirus disease 2019?. Nutrition.

[122] Das U.N. (2020). Can Bioactive Lipids Inactivate Coronavirus (COVID-19)?. Arch. Med. Res..

[123] Rogero M.M., Leão M.D.C., Santana T.M., Pimentel M.V.D.M.B. (2020). Potential benefits and risks of omega-3 fatty acids supplementation to patients with COVID-19. Free Radic. Biol. Med. J..

[124] Sorokin A.V., Karathanasis S.K., Yang Z.H., Freeman L., Kotani K., Remaley A.T. (2020). COVID-19—Associated dyslipidemia: Implications for mechanism of impaired resolution and novel therapeutic approaches. FASEB J..

[125] Pham T.L., He J., Kakazu A.H., Calandria J., Do K.V., Nshimiyimana R., Lam T.F., Petasis N.A., Bazan HE P., Bazan N.G. (2021). ELV-N32 and RvD6 isomer decrease pro-inflammatory cytokines, senescence programming, ACE2 and SARS-CoV-2-spike protein RBD binding in injured cornea. Sci. Rep..

